# Transgenic Tobacco Plants Expressing Synthetic Peptides: A Functional and Structural Analysis for Pathogen Resistance

**DOI:** 10.1111/pbi.70287

**Published:** 2025-09-15

**Authors:** Karishma Biswas, Sudipta Mitra, Dibakar Roy, Sanhita Roy, Dibakar Sarkar, DeokHyun Son, Rohit Das, Anuradha Roy, Dulal Senapati, Humaira Ilyas, A. Harikishore, Ranjit Biswas, Suman Chakrabarty, DongKuk Lee, Indranil Biswas, Sudipto Saha, Pallob Kundu, Anirban Bhunia

**Affiliations:** ^1^ Department of Chemical Sciences, Unified Academic Campus Bose Institute Kolkata India; ^2^ L V Prasad Eye Institute Hyderabad India; ^3^ Chemical, Biological and Macromolecular Sciences S. N. Bose National Centre for Basic Sciences Kolkata India; ^4^ Department of Biological Sciences, Unified Academic Campus Bose Institute Kolkata India; ^5^ Department of Fine Chemistry Seoul National University of Science and Technology Seoul Korea; ^6^ Chemical Sciences Division Saha Institute of Nuclear Physics Kolkata India; ^7^ School of Biological Sciences Nanyang Technological University Singapore Singapore; ^8^ Department of Microbiology University of Kansas Medical Center Kansas City Kansas USA

**Keywords:** antimicrobial peptides, lipopolysaccharide (LPS), multidrug resistance, nuclear magnetic resonance (NMR), saturation transfer double difference (STDD), transferred NOESY (trNOESY), transgenic plant

## Abstract

The emergence of multidrug‐resistant pathogens poses a serious threat to human health and agriculture. Current antimicrobial strategies against phytopathogens are often ineffective, failing to ensure food security while contributing to environmental pollution. Synthetic antimicrobial peptides (AMPs) offer a promising alternative due to their broad‐spectrum activity and potential for recombinant production. In this study, we investigated the antibacterial potential of two synthetic peptides, VR18 and KG18, against animal as well as plant phytopathogens. Both peptides showed selective binding to bacterial membranes, while exhibiting no toxicity or allergenicity in animal cells. Using solution‐state NMR, we explored how their structure relates to their function in disrupting bacterial membranes. When expressed transgenically in *Nicotiana tabacum* , VR18 and KG18 conferred resistance to *Pseudomonas syringae* pv. *tabaci*—a significant plant pathogen—without interfering with the plant's normal stress responses or metabolic activity. These results underscore the potential of AMPs as a sustainable, in vivo alternative to traditional antimicrobials in agriculture and open the door to broader applications in managing phytopathogenic threats.

## Graphical abstract

Peptide Power: VR18 & KG18 Shield Tobacco from Bacterial Attack. A schematic shows how antimicrobial peptides VR18 and KG18 fight Pseudomonas syringae—bursting bacterial membranes and boosting disease resistance in transgenic tobacco, without harming plant health.

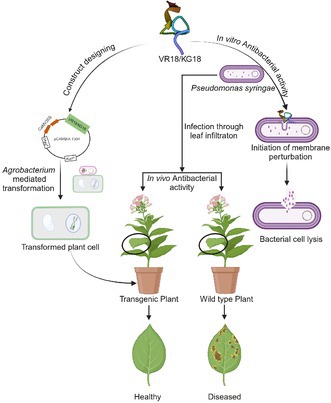

## 1 Introduction

Crop diseases caused by phytopathogens significantly reduce the quality and yield of agricultural products, resulting in substantial economic losses and burdening farmers globally (Savary et al. 2019). Current control methods rely heavily on chemical pesticides and antimicrobials, which pose significant risks to human health and the environment. The United States Environmental Protection Agency has documented adverse effects associated with pesticide exposure, including neurotoxicity from organophosphates and carbamates, skin and eye irritation, an increased risk of chronic diseases such as cancer and diabetes, respiratory conditions like asthma, reproductive disorders, and neurodegenerative diseases such as Alzheimer's and Parkinson's (Zhou et al. 2025). These chronic disorders are often attributed to pesticides interfering with ion channels and enzymes, disturbing cellular homeostasis (Mostafalou and Abdollahi 2013).

In response, several developed countries have implemented stringent regulatory changes for pesticide registration, demanding a half reduction of the harmful ingredients, while promoting retention of the active components that are target‐specific, minimally cytotoxic, and eco‐friendly (Handford et al. 2015). These regulations have led to the banning of many pesticides, complicating efforts to manage plant diseases affecting economically significant crops.

Plant genetic engineering, including conventional transgenic approaches as well as, genome‐editing technologies has emerged as a powerful tool for the improvement of crop health (Dong and Ronald 2019; Shi et al. 2017; Wang et al. 2025). RNA interference (RNAi), for example, disrupts viral life cycles by triggering double‐stranded RNA (dsRNA)‐mediated degradation of viral RNA and has been widely used to cultivate virus‐resistant crops, such as transgenic Cucurbita and *Carica papaya* , which have been successfully grown in the United States for over 25 years (Jia et al. 2017). Other methods employ CRISPR‐Cas technologies employing Cas nucleases to generate sequence‐specific ribonucleoproteins that target and degrade viral DNA or RNA (Wright et al. 2016). The expression of resistance (R) genes, based on the “gene‐for‐gene” theory, has also shown potential, as demonstrated by the expression of the pepper *Bs2* R gene in tomato plants, which conferred resistance against bacterial spot disease caused by *Xanthomonas sp* (Flor 1971; Horvath et al. 2013). Despite these advancements, these strategies are often limited by their narrow microbial spectrum, underscoring the need for broader, more versatile approaches (Dong and Ronald 2019).

Antimicrobial peptides (AMPs) have been a part of the innate immune system of organisms ranging from microorganisms and invertebrates to higher plants and animals (Pasupuleti et al. 2012; Roy et al. 2024). They exhibit a rapid mode of action as well as broad‐spectrum activity against bacteria, fungi, viruses, and parasites (Hancock 1997). Compared to most existing antimicrobial agents, AMPs display unspecialised modes of action mainly perturbing the negatively charged microbial membranes. Alteration of the microbial membrane composition is a highly energetically challenging process (Andersson et al. 2016). Some AMPs have also been reported to have intracellular targets, such as indolicidin, a peptide targeting the cytoplasmic membrane as well as inhibiting DNA synthesis (Ghosh et al. 2014). Such multi‐modal mechanisms of action make the development of resistance against these AMPs difficult, thus opening a new horizon for investigating AMPs as the next‐generation weapon for tackling drug‐resistant microbial pathogens. However, in recent times, there have been few reports of AMP resistance (LaRock and Nizet 2015). Most naturally occurring AMPs also display host cytotoxicity in higher concentrations, limiting their use to only topical applications (Hancock et al. 1995). Peptide designing strategies using modified analogues of naturally occurring AMPs as well as synthetically designed peptides have thus gained immense attention with a view to developing newer antimicrobial agents with desired properties (Gordon et al. 2005).

AMPs have garnered huge interest as potential candidates for combating crop diseases, owing to their versatile biological properties. Plant AMPs are primarily classified based on sequence similarity, cysteine motifs, and tertiary structure (Tam et al. 2015). Most plant AMPs are positively charged at physiological pH and range from 2 to 10 kDa in their molecular weight (Li et al. 2021). Among them, cationic AMPs (CAMPs) are the most studied, owing to their broad‐spectrum activity at low concentrations, unique mechanisms of action, and the difficulty of microorganisms to develop resistance against them (Chen and Jiang 2023; Hancock and Rozek 2002; Zhang et al. 2001).

Interestingly, the structure of AMPs has been a major driving force in their functional activity. Some plant AMPs, like plant defensins and thionins, exhibit β‐pleated structures whereas others, like the α‐hairpinin family, contain two anti‐parallel helices (Li et al. 2021). Natural plant AMPs are usually constitutively expressed at basal levels until induced by pathogen attack, leading to simultaneous production and accumulation of multiple AMPs in various plant parts (Mith et al. 2015). The expression of heterologous AMPs in transgenic plants has opened promising avenues for developing plant disease resistance. Radish defensin Rs‐AFP2, for example, in tomato and tobacco plants, has been shown to confer resistance against *Amantia longipes* infection (Sher Khan et al. 2019). However, despite their propitious therapeutic potential, these natural products are characterised by a limited spectrum of biological activity and restricted efficacy towards specific targets, demanding further research for expanded utility.

Expression of intact AMPs in plants encounters several challenges, like vulnerability to endogenous proteolytic cleavage, adverse effects on plant membranes, and reduced efficacy. Addressing these obstacles is essential for optimising AMP‐based strategies in agricultural applications. In this study, we have adopted an alternate strategy overexpressing two rationally designed AMPs, namely VR18 (VARGWGRKCPLFGKNKSR) and KG18 (KNKSRVARGWGRKCPLFG) in *Nicotiana tabacum* . These peptides were non‐cytotoxic to mammalian cells and exhibited broad‐spectrum activity against a wide range of animal and plant pathogens. Biophysical analyses further demonstrated that the peptides exert their antimicrobial effects through interactions with both the outer‐ and inner‐membrane lipid components of bacteria. Importantly, these peptides did not perturb the plant model membrane mimic and were stable in plant extract containing proteases. A combination of molecular biology and metabolomics studies demonstrated that the endogenous expression of these peptides in transgenic plants strengthens the plants' innate defence mechanisms without disrupting their metabolic processes.

## 2 Results

### 2.1 Peptide Designing

**FIGURE 1:**
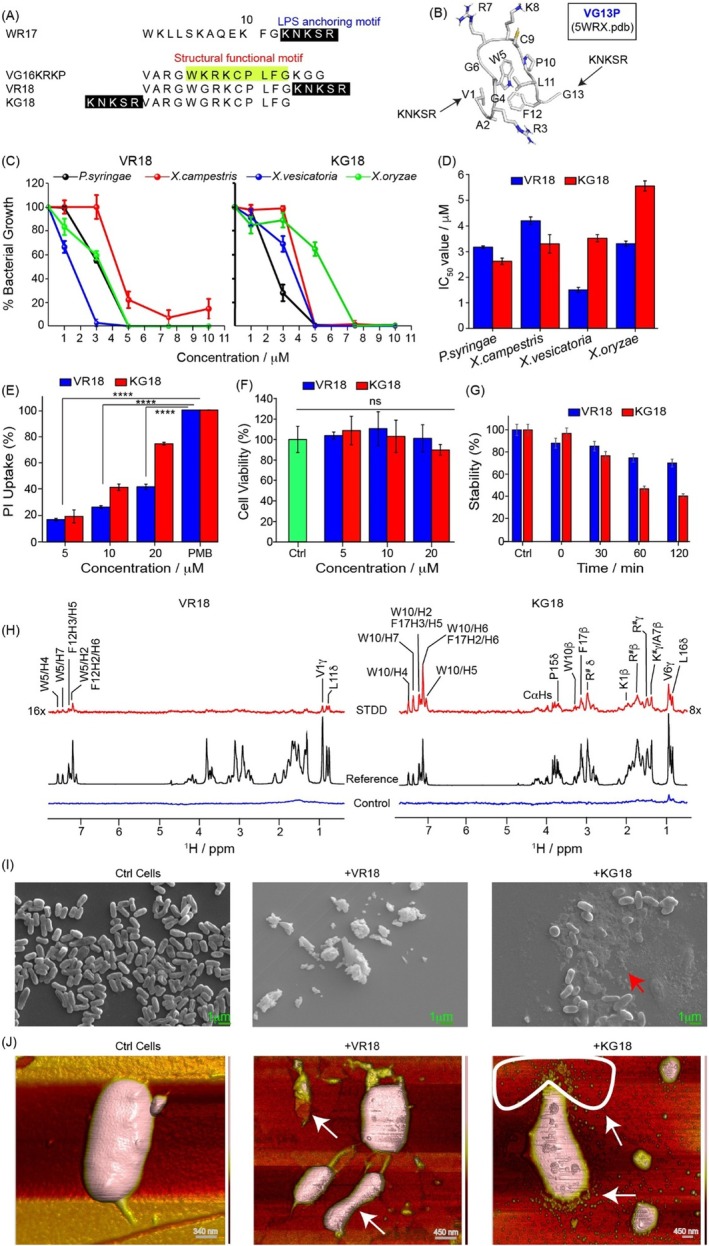
Rational design strategies for VR18 and KG18 and their characterisation against phytopathogens. (A) The primary amino acid sequences of peptides VR18 and KG18 were developed through rational design, integrating the LPS anchoring motif from WR17 (highlighted in black) with the structural motif of VG16KRKP (highlighted in yellow). Since the K14‐G15‐G16 motif of VG16KRKP demonstrates flexibility and is not critical for stability, a shorter analog, VG13P, was created. Finally, VR18 and KG18 were synthesised by attaching the KNKSR motif from WR17 to either the C‐terminal or N‐terminal of VG13P. (B) Solution‐state NMR structure of VG13P in the presence of LPS (PDB accession code: 5WRX). (C) Bacterial growth inhibition assays demonstrated that VR18 and KG18 effectively inhibited several plant pathogens. (D) The half‐maximal inhibitory concentration (IC_50_) values for VR18 and KG18 were determined from these bacterial growth inhibition assays. (E) Membrane permeabilization of *Pseudomonas syringae* within 30 min of treatment with VR18 and KG18 was assessed using propidium iodide (PI) dye uptake. Untreated cells served as negative control and did not allow the permeation of PI, showing intact membrane, whereas PMB‐treated cells served as positive control and showed maximum PI uptake. (F) The MTT cell viability assay was conducted on HEK293 cells treated with varying concentrations of VR18 and KG18 for 24 h to evaluate cytotoxic effects. Untreated cells served as control. (G) The proteolytic stability of VR18 and KG18 in tobacco plant extract containing proteases was analysed using reverse‐phase HPLC, revealing a half‐life exceeding 60 min. Data in panels (D) through (G) are presented as mean ± SEM, with *n* = 3. Statistical significance is indicated by *, representing *p* ≤ 0.05 according to a two‐tailed Student's *t*‐test, while “ns” denotes non‐significant results. (H) Saturation transfer double difference (STDD) spectra provided residue‐specific binding information, capturing how VR18 and KG18 interact with *Pseudomonas syringae* cells at an atomic resolution. (I) Scanning electron microscopy (SEM) images illustrated that control cells without peptide treatment remained intact. However, exposure to VR18 and KG18 resulted in a complete loss of cellular integrity at a concentration of 1× MIC_99%_. (J) Atomic force microscopy (AFM) analysis of single cells of *Pseudomonas syringae,* not treated with VR18 and KG18 showed no discernible morphological changes. In contrast, treatment at a concentration of 1× MIC_99%_ led to observable membrane damage and the release of intracellular materials.

We previously designed and characterised the synthetic peptide VG16KRKP (VARGWKRKCPLFGKGG) and evaluated its antimicrobial activity against a range of Gram‐negative bacterial pathogens, including both plant‐ and animal‐associated strains, as well as animal fungal pathogens. VG16KRKP exhibited broad‐spectrum antimicrobial activity but notably lacked efficacy against the Gram‐negative pathogen *Pseudomonas aeruginosa* and Gram‐positive bacteria (Datta et al. 2015). Structural analysis using high‐resolution NMR, combined with mutational studies, identified residues W5, L11, and F12 as critical for bioactivity (Figure 1A). These residues formed a hydrophobic hub essential for membrane interaction, and substitution of any of these residues resulted in significantly reduced or complete loss of antifungal efficacy (Mohid, Biswas, et al. 2022).

Lipopolysaccharide (LPS) is a major component of the outer membrane of Gram‐negative bacteria and is an endotoxin (Rietschel et al. 1994). LPS is not only responsible for the antigenic property of the bacterial cell but also contributes to the structural integrity of the cell membrane. It serves as the first line of defence for the bacterial cells by stabilising the outer leaflet of the cell membrane and preventing the penetration of several antimicrobials (Zhang et al. 2013). Hence, to enhance activity against *P. aeruginosa* , we engineered two chimeric peptides, KG18 and VR18, by fusing the structural‐functional domain of a truncated VG16KRKP variant (VG13P: WKRKCPLFG) (Datta et al. 2017) with the LPS‐binding motif derived from the WR17 peptide (Figure 1A,B). WR17 (WKLLSKAQEKFGKNKSR), a cationic antimicrobial peptide originally developed from a lactoferrampin sequence, was previously shown to interact effectively with LPS, disrupting its barrier function (Ghosh et al. 2014). Incorporating this LPS‐binding motif (K^13^NKSR^17^ of WR17) was intended to facilitate outer membrane penetration and enhance bactericidal activity. Both KG18 and VR18 (Figure S1) exhibited significantly improved antimicrobial activity against *P. aeruginosa* , including both planktonic and biofilm forms (Mohid et al. 2019).

### 2.2 VR18 and KG18 Displayed Potency Against Plant Pathogens

We further assessed the antimicrobial efficacy of KG18 and VR18 against both animal pathogens from the ESKAPE group and several agriculturally significant plant pathogens, including *Xanthomonas campestris* pv. *vesicatoria* (Potnis et al. 2015), *X. campestris* pv. *campestris* (Alvarez 2000), *X. oryzae* pv. *oryzae* (Naqvi 2019), *and Pseudomonas syringae* pv. *tabaci* (Ramegowda et al. 2013) (Figure S2). These pathogens are known to severely impact commercially important crops (Figure 1C). Both peptides were highly effective, completely inhibiting the growth of the Gram‐negative pathogens with minimal inhibitory concentrations (MIC_99%_) ranging from 3 to 10 μM (Figure S3). Notably, the antimicrobial potency of KG18 and VR18 was significantly greater than that of the original VG16KRKP peptide. The half‐maximal inhibitory concentration (IC_50_) values for VR18 ranged from 1.50 to 4.25 μM, while for KG18, the range was 2.75 to 5.50 μM (Figure 1D).

To assess the membrane integrity (Mohid et al. 2019) and the potential mechanisms of peptide interaction with the plant pathogen *P. syringae* pv. *tabaci*, we employed the membrane‐impermeable nucleic acid stain, propidium iodide (PI). Under physiological conditions, both AMPs, KG18 and VR18, caused membrane disruption in *P. syringae* , indicated by increased fluorescence intensity of PI. KG18 demonstrated a stronger effect, resulting in approximately 75% uptake of PI, while VR18 showed only 40% uptake (Figure 1E). The positive control, polymyxin B (PMB), displayed the maximum PI uptake. Collectively, these results indicate that KG18 is a more effective membrane disruptor of *P. syringae* compared to VR18.

### 2.3 Cell Viability, Allergenicity and Stability of VR18 and KG18

Next, we evaluated the cytotoxic effects of VR18 and KG18 on human embryonic kidney (HEK 293) cell lines; both peptides exhibited no toxicity even at concentrations as high as 4× MIC_99%_ (Figure 1F). We assessed the predictability of the protein/peptide to generate allergenicity as per WHO and FAO guidelines. Using the AlgPred server (http://www.imtech.res.in/raghava/algpred/) (Saha and Raghava 2006), which uses a support vector machine (SVM) algorithm (Table S1), we found that both the AMPs were non‐allergenic (Table S1). Additionally, the ADMET properties of VR18 and KG18 were analysed using the ADMET‐AI server (https://admet.ai.greenstonebio.com) (Swanson et al. 2024). Machine learning (ML) predictions indicated that both peptides are non‐toxic, exhibit low oral absorption, do not cross the blood–brain barrier, and are non‐toxic to the hERG channel (Figure S4).

Additionally, we also investigated the stability of VR18 and KG18 in tobacco plant extract, which contains active plant proteases. VR18 displayed a half‐life of over 120 min, while the half‐life of KG18 was more than 60 min (Figure 1G). Taken together, these findings clearly indicate that the peptides are non‐toxic, non‐allergenic, and stable in the presence of plant proteases, highlighting their potential for development as antimicrobials, particularly in agricultural applications.

### 2.4 Mapping of VR18 and KG18 Interaction With *P. syringae* at Atomic Levels

Since both the peptides VR18 and KG18 displayed antibacterial activity against *P. syringae* , we aimed to further investigate the molecular interaction with the bacterial cells at an atomic resolution using solution‐state live cell NMR spectroscopy. To achieve this, we employed saturation transfer double difference (STDD) NMR to map the key peptide residues that are involved in the interaction with the live cells. The STDD signals for VR18 were predominantly observed for the aromatic ring protons of residues W5 and F12 as well as the γ‐ and δ‐ methyl protons of V1 and L11, respectively. These findings suggest that these specific residues play a direct and active role in membrane binding and interactions with the cell surface.

In contrast, the aromatic ring protons of W10 and F17, along with the γ‐ and δ‐ methyl protons of V6 and L16, exhibited the strongest STD signal. This suggests that these hydrophobic residues are situated near the bacterial cellular surfaces. Additionally, moderate STD signals were observed from β‐, γ‐ and δ‐ protons of Arg and Lys residues (K1/K3/R5/R8/R12/R13), indicating that these positively charged residues are in close proximity to bacterial surfaces. Weak STD peaks were also detected for P10, β‐ protons of A7 as well as certain aromatic residues, suggesting a secondary involvement in the interaction (Figure 1H).

### 2.5 Visualisation of the Effect of AMP on *P. syringae* Cells

Next, we visualised the effect of the peptides on *P. syringae* cells using scanning electron microscopy (SEM). In the absence of the peptides, bacterial cells remained intact and maintained a distinct rod‐shaped morphology (Figure 1I). However, treatment of the cells with both peptides at a concentration of 0.5× MIC_99%_ led to significant clumping of the cells, indicative of a stress‐induced response, along with a reduction in the overall bacterial cell population (Figure S5A). Strikingly, at higher concentrations (1× and 2× MIC_99%_), the cells were completely disrupted, displaying no distinguishable cellular morphology, with remnants of cellular debris observed following lysis (Figure 1I and Figure S5A). To further probe the effect of the peptides on the membrane surface, we conducted high‐resolution solid‐state atomic force microscopy (AFM). The results revealed a drastic cell shrinkage accompanied by membrane blebbing at concentrations of 1× MIC_99%_ and 2× MIC_99%_ of the peptides (Figure 1J and Figure S5B). Additionally, we observed significant surface contortion, irreversible damage, fragmentation, and release of intracellular components from the bacterial cells. Collectively, these microscopic techniques unravelled the bacteriolytic effect of the peptides.

### 2.6 Transgenic Plants Development Expressing VR18 and KG18 Peptides

**FIGURE 2:**
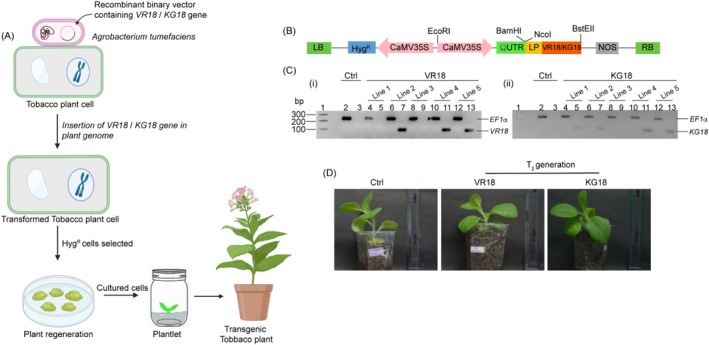
Expression of VR18 and KG18 genes in transgenic *Nicotiana tabacum* . (A) Illustration of Agrobacterium‐mediated transformation of tobacco plant cells with the VR18/KG18 vector. Transgenic plant calli were regenerated on selective media containing Hygromycin, followed by differentiation and growth into healthy plants, created using BioRender.com. (B) A schematic representation of the plant expression vector, pCAMBIA 1304, is provided, detailing the expression of the VR18 and KG18 encoding genes in tobacco. The genes are driven by the CaMV 35S promoter, and the positioning of the Hygromycin resistance gene (*Hyg* ^R^) is indicated. LB and RB denote left border and right border, respectively. ΩUTR is *Tobacco etch virus* derived 5′ UTR required for the regulation of translation. LP denotes the coding region of the leader peptide derived from the DmAMP1 precursor, which facilitates the secretion of the VR18 and KG18 peptides in the apoplastic region. NOS depicts 3′‐untranslated terminator region of the nopaline synthase gene of *Agrobacterium tumefaciens* . The scheme is not drawn in scale. (C) Reverse Transcription PCR (RT‐PCR) results for expression of VR18 and KG18 across five independent transgenic lines are shown. The agarose gel image shows successful amplification of the VR18 amplicon in three transgenic lines (lane 7,11,13) and the KG18 amplicon in four lines (lane 5,7,11,13). These positively identified lines were selected for further experimental analysis, while wild‐type plants displayed no amplification of both VR18 or KG18 amplicons. The expression of *EF1*α gene was checked to verify the sample integrity (lanes 2, 4, 6, 8, 10, and 12). (D) Photograph showcases 1‐month‐old control and transgenic tobacco plants from T_2_ generation, both of which exhibit similar phenotypic morphology.

We conducted *Agrobacterium tumefaciens* ‐mediated transformation using the pCAMBIA 1304 expression vector (Figure 2A,B), resulting in the generation of five primary transgenic lines of *Nicotiana tabacum*, each expressing the VR18 and KG18 genes from five different callus cultures. These transgenic lines were maintained through the T_1_ generation, with selection based on hygromycin resistance. Notably, the transgenic plants were phenotypically indistinguishable from wild‐type plants grown in the glasshouse (Figure 2D).

The expression of VR18 and KG18 transcripts in the T_1_ transgenic plants was confirmed using RT‐PCR. Three distinct lines exhibited expression of the VR18 transgene, indicated by an 88 bp amplicon (Figure 2C, left panel, lanes 7,11,13), while expression of the KG18 transgene was detected in four transgenic lines, evidenced by a 113 bp amplicon (Figure 2C, right panel, lanes 5,7,11,13). These transgenic lines were further maintained through the T_2_ generation for subsequent studies. Contrastingly, peptide‐specific RNA products were not amplified from the control plant samples (Figure 2C lane 3) or from vector control plants (Figure S6). However, expression of the housekeeping *EF1*α gene was detectable in all tested samples. Additionally, we also tested the translation of these peptides in the transgenic plants using MALDI analysis. The spectra of plant extracts from the transgenic plants showed a significant peak in the m/z range of 2063 Da and 2066 Da in the case of VR18 and KG18, respectively, corresponding to the molecular weight of the peptides (Figure S7). No such peak was observed in the spectra of the plant extracts from the vector control plants.

### 2.7 VR18 and KG18 Transgenic Plants Show Strong Resistance to a Phytopathogen

**FIGURE 3:**
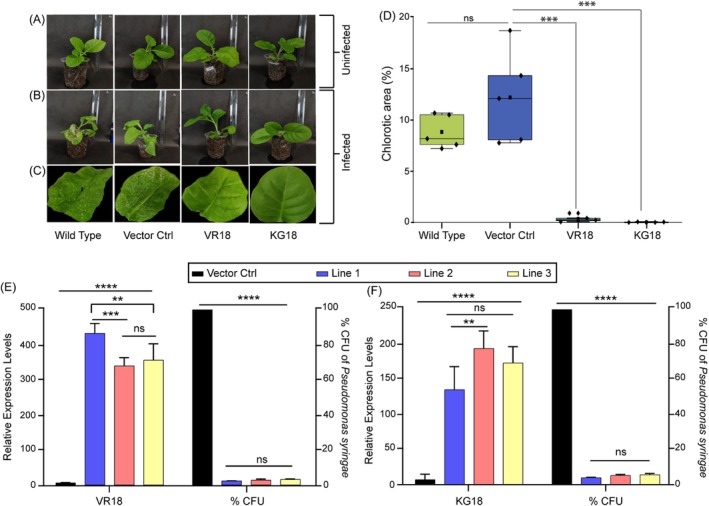
In vivo analysis of the antibacterial activity of VR18 and KG18 against *P. syringae* pv. *tabaci*. (A) The photographs display 1‐month‐old uninfected wild‐type plants, showcasing their healthy appearance prior to any pathogen exposure. (B) In contrast, the images depict wild‐type plants infected with *Pseudomonas syringae* pv. *tabaci* for 72 h, highlighting observable infection symptoms. (C) Leaf photographs reveal the wild‐type, vector control, and transgenic lines expressing VR18 and KG18. The transgenic lines exhibit no significant chlorosis or infection symptoms, whereas the wild‐type and vector control plants show extensive chlorosis and stunted growth after 72 h of infection. (D) A comparative analysis of lesion areas post‐infection across wild‐type, vector control, and transgenic leaves expressing VR18 and KG18 is presented (*n* = 5). Significant changes are marked with * (*p* ≤ 0.05) according to a two‐tailed Student's t‐test, while “ns” indicates non‐significance. (E) Bar graph showing the transcript levels of the VR18 expressing gene in vector control plants, as well as three different lines of VR18 expressing transgenic plants, and the % bacterial CFU count in all three lines post 72 h of infection compared to the vector control plants. (F) Bar graph showing the transcript levels of the KG18 expressing gene in vector control plants, as well as three different lines of KG18 expressing transgenic plants and the % bacterial CFU count in all three lines post 72 h of infection compared to the vector control plants. Each column represents the mean of three biological replicates, with error bars indicating SEM. Significant changes are marked with * (*p* ≤ 0.05) according to a two‐way ANOVA analysis, while “ns” indicates non‐significance. This analysis underscores the protective effect of the transgenic expression of VR18 and KG18 in mitigating disease advancement compared to the controls.

We evaluated the in vivo activity of the peptides by challenging both wild‐type and transgenic plants (Figure 3A) with *P. syringae* pv. *tabaci*. *Pseudomonas*‐infected wild‐type and vector control leaves developed numerous small brown spots throughout the leaf surface within 72 h, and in some cases, a chlorotic halo also appeared around the infection sites (Figure 3B,C). After 96 h of infection, we observed a pronounced chlorosis and complete wilting of the leaves in the control plants, leading to stunted growth with prolonged infection. In contrast, VR18 transgenic plants showed only a few small brown spots in some leaves, and by 96 h of post‐infection, no further progression of disease symptoms was observed, with the overall health of the plants remaining intact. Notably, no visible disease symptoms were present in the KG18 transgenic plants even after 96 h of post‐infection (Figure S8). Quantitative assessment of the lesion area revealed a remarkable reduction in disease progression with a decrease of 96% for VR18 and 99% for KG18 transgenic leaves (Figure 3D). These findings strongly indicate that both VR18 and KG18 peptides displayed bioactivity that effectively suppressed the disease onset by inhibiting pathogen invasion in *N. tabacum* . Furthermore, we quantified the colony‐forming unit of *P. syringae* post 96 h of infection in the leaf extracts of vector control plants and transgenic plants. The bacterial CFU count clearly showed a reduction of 85%–90% in the case of the transgenic plants compared to the vector control plants (Figure S9).

We also assessed the impact of different levels of VR18 and KG18 expression across the three different lines studied on the CFU count post‐infection. Interestingly, although the expression of VR18 was higher in Line 1 compared to the expression in the other two lines, the CFU count was similar in all three VR18‐expressing lines. This indicates that the constitutive production of the peptides was above the threshold level in all lines to inhibit the infection of *P. syringae* (Figure 3E). Similar results were obtained in the case of the KG18‐expressing plants. KG18 expression was slightly higher in Line 2 compared to the other two lines. However, the difference in CFU count across the three lines was negligible (Figure 3F).

### 2.8 ROS Accumulation in *P. syringae* Infected Transgenic Plants

**FIGURE 4:**
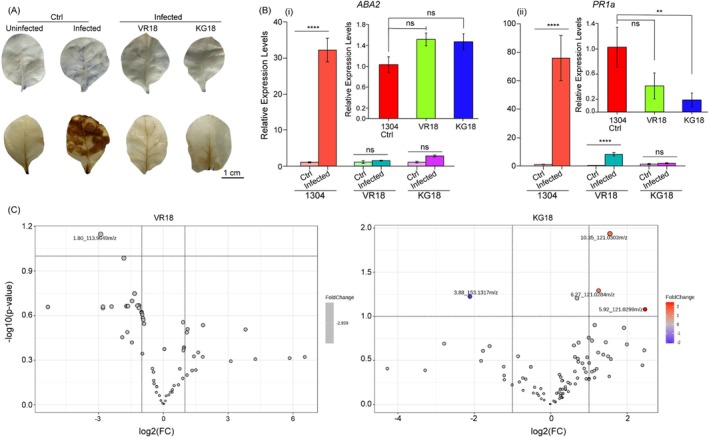
Assessment of AMP transgenics at the molecular level. (A) NBT and DAB staining of 1‐month‐old vector control and transgenic plants was performed to detect ROS generation following infection with *P. syringae* . Scale bars represent 1 cm. The blue coloration resulting from NBT staining and the brown precipitate from DAB staining indicate extensive ROS generation in infected control plants, suggesting a significant production of superoxide and hydrogen peroxide (H_2_O_2_) compared to transgenic plants. (B) (i) The transcript levels of the abiotic stress response gene, *ABA2*, were measured after infection with *P. syringae* . qRT‐PCR (RT‐PCR) analysis of RNA extracted from 1‐month‐old vector control and transgenic plants demonstrated a significant upregulation in the relative expression levels of *ABA2* in control plants. The inset provides a visual representation of the transcript levels of *ABA2* in both the vector control and transgenic lines. (ii) Transcript levels of the biotic stress response gene, *PR1a*, were also analysed post‐infection. The qRT‐PCR results indicated notable upregulation of *PR1a* in control plants, while plants expressing VR18 showed only a slight increase in expression compared to the vector control. The inset illustrates *PR1a* transcript levels in both groups. The expression levels of these genes were normalised to *EF1*α. Each column represents the mean of three biological replicates, with error bars indicating SEM. Significant changes are marked with * (*p* ≤ 0.05) according to a two‐tailed Student's t‐test, while “ns” indicates non‐significance. (C) A volcano plot was generated to analyse metabolites from the VR18 versus control and KG18 versus control groups following metabolite screening using LC–MS/MS and analysis with Progenesis QI. No significant differences in metabolite expression were observed in VR18 or KG18 plants relative to the control. At a *p*‐value > 0.05 (false discovery rate not adjusted) and fold change > 2, three compounds with m/z approximately 121 (differing in retention times) were upregulated in KG18 plants, while one compound with m/z approximately 153 (retention time of 3.88 min) was downregulated. Each dot on the plot represents a compound ID, with the corresponding retention time (in minutes) indicated alongside its mass. All LC–MS/MS experiments were performed with biological triplicates in positive ion mode.

Phytopathogen infection can disrupt ROS balance, leading to elevated ROS levels that suppress the cell's ability to maintain redox equilibrium (Sahu et al. 2022). To evaluate the ROS generation in transgenic plants following *P. syringae* attack, we used a histochemical staining method to monitor the generation of superoxide (O_2_^−^) and hydrogen peroxide (H_2_O_2_), the major components of ROS along side hydroxyl ions. As expected, the infected vector control leaves displayed a blue colour upon nitro blue tetrazolium (NBT) staining, indicative of diformazan production at the sites of O_2_^−^ generation. However, no such colour change was observed in the leaves of transgenic plants post‐infection (Figure 4A).

We further utilised a leaf staining assay with 3,3^−^‐diaminobenzidine (DAB), which forms brown precipitates through H_2_O_2_‐mediated polymerisation. Consistent with our previous findings, extensive brown coloration was observed in the infected vector control leaves while no brown staining was detected in the infected transgenic leaves (Figure 4A). Taken together, these results suggest that the transgenic plants effectively manage ROS levels in response to pathogen challenge.

### 2.9 Expression of Stress Genes Remains Unchanged in AMP Transgenic Plants During Infection

Plant health and growth are significantly affected by the abiotic and biotic stresses they encounter in their natural environments (Nawaz et al. 2023). To assess the impact of VR18 and KG18 expression on plant health, we measured the expression levels of two hallmark genes: *ABA2*, which catalyses the final step of abscisic acid biosynthesis and serves as an indicator of abiotic stress response; and *PR1a*, a pathogenesis‐related gene indicative of biotic stress response, in three different transgenic lines expressing each peptide. Our results indicated a slight but statistically insignificant upregulation of *ABA2* expression in all three transgenic lines compared to the vector control plants (Inset, Fig. 4Bi). Conversely, *PR1a* gene expression exhibited a slight downregulation across all transgenic lines, with more pronounced downregulation observed in KG18‐expressing lines compared to the VR18 lines (Inset, Figure 4Bii). To further assess the plant's responses, we analysed the relative expression levels of the *ABA2* and *PR1a* genes using total RNA extracted from control and infected leaves distal to the infection site. In the vector control leaves, we noted a significant upregulation of both *ABA2* and *PR1a* expression levels (Figure 4B and Figure S10). Interestingly, *ABA2* expression levels remained unchanged in both transgenic lines upon pathogen infection (Figure 4Bi and Figure S10Ai,ii). Moreover, *PR1a* expression was consistently lower in both transgenic plants; while there was a slight upregulation in the VR18‐expressing plants, this change was insignificant in KG18‐expressing lines. Nevertheless, during infection, *PR1a* expression in both transgenic lines was at least 8‐fold downregulated compared to control plants (Figure 4Bii and Figure S10Bi,ii). These observations are consistent with the infection study findings, where VR18‐expressing plants showed some symptom development, whereas KG18 plants did not.

### 2.10 Peptide Expression Exhibited Minimal Impact on the Secondary Metabolite Profile of Tobacco

Although the intrinsic expression of VR18 or KG18 peptides resulted in negligible abiotic and biotic stress responses in the tobacco plant, it is essential to examine the impact of these peptides on the regulation of plant cellular metabolites, as they are not naturally part of the plant's defence system. To investigate this, we employed Liquid Chromatography Mass Spectroscopy (LC/MS) to analyse the metabolic changes in *N. tabacum* associated with the expression of VR18 and KG18 peptides. A total of 362 metabolites were identified based on the m/z ratio and retention time of LC/MS (Table S4).

We generated two volcano plots to compare the metabolite profiles between the control group and the KG18 plants, as well as between the control and VR18 plants (Figure 4C). Our analysis revealed no significant upregulation or downregulation of the screened metabolites in the plants expressing VR18 and/or KG18 when compared to the control groups. However, at a *p*‐value threshold greater than 0.05, we observed that certain compounds with an m/z~121 and varying retention times were notably upregulated in the KG18‐expressing plants, with a fold change greater than 2. These upregulated metabolites were primarily identified as polyphenols, including 3‐Hydroxybenzoic acid, protocatechuic aldehyde, and 4‐Hydroxybanzaldehyde etc. (Table S5). These phenolic compounds are recognised for their antioxidant and antimicrobial properties (Srivastava et al. 2023).

On the other hand, we found downregulation in metabolites from the terpene group (m/z~153; retention time of 3.88 min) in the KG18‐expressing plants. In the comparison between the control and VR18‐expressed transgenic plants, no changes in relative metabolite expression were observed. Overall, metabolome profiling indicated that the transgenic expression of exogenous VR18 and KG18 did not significantly affect plant metabolism or the production of bioactive natural compounds (Table S6). Collectively, these results suggest that the transgenic expression of these peptides may confer resistance to pathogens primarily through peptide‐mediated disruption of pathogen membranes rather than through significant changes in metabolic pathways.

### 2.11 Interaction of AMPs with Bacterial Lipopolysaccharides

**FIGURE 5:**
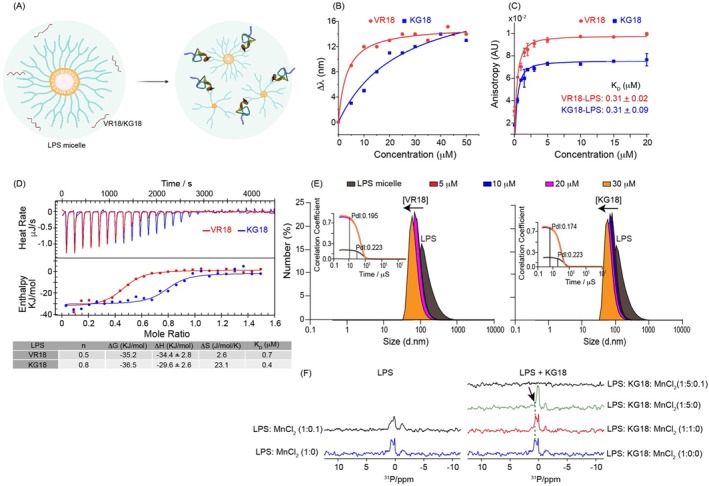
Biophysical studies of VR18 and KG18 interaction with LPS. (A) Illustration depicts how VR18 and KG18 interact with an LPS micelle, created using BioRender.com. The larger molecular structures of LPS are broken down into smaller forms, while the random coil peptides in solution adopt more structured conformations upon their interaction with LPS. (B) Fluorescence emission shifts of tryptophan (λ_max_) in VR18 and KG18 in the presence of LPS at various peptide ratios. The observed hypsochromic shift indicates a direct interaction between the peptides and LPS. (C) Anisotropy values of VR18 and KG18 displayed significant changes with increasing LPS concentration. The dissociation constant (K_D_) values calculated for the peptides bound to LPS demonstrated strong interactions. (D) Isothermal titration calorimetry (ITC) experiments involving VR18 and KG18 with LPS micelles revealed exothermic reactions occurring over time. The lower panels show enthalpy change per peptide injection plotted against the molar ratio of VR18/KG18 to LPS, while the accompanying table lists thermodynamic binding parameters at 310 K. The variable “n” indicates the binding stoichiometry. (E) Dynamic light scattering (DLS) of monodisperse LPS micelles titrated with VR18 and KG18 indicated significant changes in size and shape. This is evidenced by a decrease in polydispersity index (PDI) and differences in delay time (illustrated by the black dotted line). (F) NMR spectra of LPS were acquired with increasing concentrations of KG18 in the presence of MnCl_2_, a quenching agent. The results showed an increase in the intensity of the LPS phosphate head groups and a notable shift. It was observed that MnCl_2_ did not significantly affect the LPS peaks in the absence of KG18, but in the presence of KG18, the peaks became completely quenched. Each experiment was performed in triplicate.

To investigate the binding interaction of peptides VR18 and KG18 with lipopolysaccharide (LPS), a key component of the outer membrane of Gram‐negative bacteria (Carnicelli et al. 2013; Hotchkiss et al. 2016), we utilised fluorescence spectroscopy, exploring the Trp residues present in both peptides. It is noteworthy to mention that AMP has to interact with LPS first before gaining access to the inner membrane (Bhunia et al. 2009). It is hypothesised that AMPs usually interact with larger aggregates of LPS micelles and cause fragmentation, adopting secondary conformations as illustrated in Figure 5A. Upon titration with increasing concentrations of LPS, the emission maxima (λ_max_) of VR18 and KG18 shifted from ~350 to 364 and 365 nm, respectively (Figure 5B). To further analyse the impact of LPS on the rotational freedom of Trp residues in the peptides, we applied fluorescence anisotropy experiments (Figure 5C). The degree of anisotropy in the presence of LPS allowed us to calculate the equilibrium dissociation constant (K_D_), which was determined to be 0.31 ± 0.02 for VR18 and 0.31 ± 0.09 μM for KG18, respectively.

Additionally, we employed isothermal titration calorimetry (ITC) experiments to measure the binding energy of both LPS‐active peptides. The results indicated that the binding reactions were spontaneous and driven by entropy, yielding dissociation constants (K_D_) of 0.70 and 0.40 μM, respectively for VR18 and KG18, which correlated well with the findings from the fluorescence techniques (Figure 5D). These analyses provide insights into the strong affinity of these peptides for LPS, highlighting their potential role in disrupting bacterial membranes.

Dynamic light scattering (DLS) measurements of LPS micelles (Ilyas et al. 2019) showed that the addition of increasing concentrations of VR18 and KG18 led to immediate alterations in the organisation of the LPS micelles. This was evidenced by a faster relaxation decay and decrease in delay time (Figure 5E inset). Consequently, we observed a decrease in the polydispersity index (PDI) from 0.22 to 0.20 for VR18 and down to 0.17 for KG18, indicating that the size distribution of the LPS aggregates became narrower following the addition of peptides. The mean hydrodynamic diameter of the LPS micelles decreased from 250 nm to 119 nm for VR18 and 114 nm for KG18 (Figure 5E).

To further investigate the mechanism of LPS aggregate disruption at an atomic resolution, we conducted one‐dimensional ^31^P solution‐state NMR experiments on LPS both alone and in the presence of increasing concentrations of KG18, using MnCl_2_ as a paramagnetic quencher (Datta et al. 2015) (Figure 5F). In these experiments, the ^31^P peaks of LPS alone were minimally affected in the presence of MnCl_2_. However, upon addition of increasing concentrations of both peptides, these peaks were completely quenched, confirming that the phosphate head groups were completely exposed to the Mn^2+^ ions. In addition, 1D ^1^H NMR spectra of LPS in the presence of KG18 and VR18 showed concentration‐dependent line broadening and chemical shift perturbation of acyl chains of LPS (Figure S11). Taken together, the findings from the DLS and ^31^P NMR experiments indicate that VR18 and KG18 interact with LPS and disrupt its structure through a detergent‐like mechanism, leading to fragmentation of LPS aggregates.

### 2.12 Interactions of AMPs with Membrane Model Mimics

**FIGURE 6:**
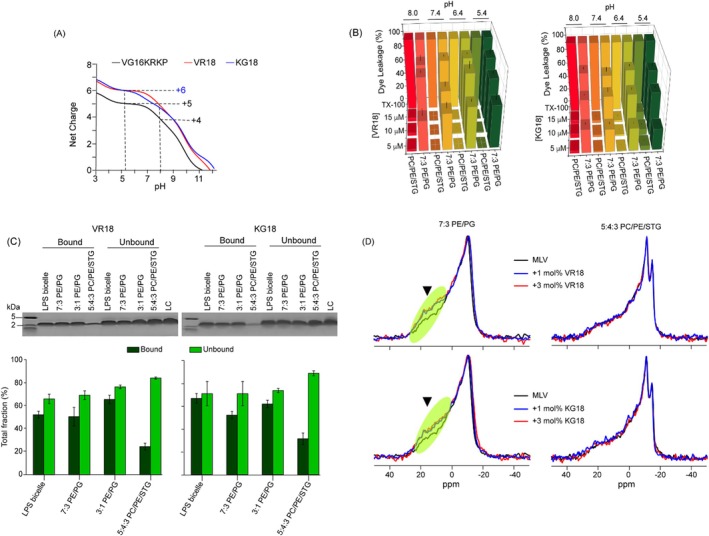
Interaction of VR18 and KG18 with model membrane mimics. (A) The net charge of the peptides VG16KRKP, VR18, and KG18 across various pH levels was calculated based on the pKa values of the constituent amino acids. This analysis provides insight into the charge characteristics of the peptides, which can influence their interactions with membrane mimics. (B) Calcein dye leakage assays were performed using vesicles composed of 7:3 POPE/POPG and 5:4:3 POPC/POPE/STG, in the presence of VR18 and KG18 at different pH levels (5.4, 6.4, 7.4, and 8.0). The results revealed that VR18 and KG18 did not significantly affect the dye leakage from the 5:4:3 POPC/POPE/STG vesicles; however, they elicited over 70% and 80% calcein release from the POPE/POPG vesicles, respectively, indicating a strong interaction with this membrane model. (C) A liposome pull‐down assay was conducted using large unilamellar vesicles (LUVs) of varying compositions, including 7:3 POPE/POPG, 3:1 POPE/POPG, 5:4:3 POPE/POPG/STG, and *Pseudomonas* LPS bicelles. After incubating the vesicles with 5 μg of each peptide and centrifugation, the bound fraction was isolated in the pellet, while the unbound fraction remained in the supernatant. This distribution was visualised using Tricine‐SDS gel electrophoresis followed by Coomassie blue staining. The intensity of the peptide bands was quantified using ImageJ and normalised against loading controls for VR18 and KG18. (D) One‐dimensional ^31^P NMR of 7:3 POPE/POPG and 5:4:3 POPE/POPG/STG multilamellar vesicles (MLVs) with and without VR18 and KG18. Increasing concentrations of the peptides led to spectral thickening in the marked area and a greater spectral range in 7:3 POPE/POPG vesicles. No significant spectral changes were observed in the 5:4:3 POPE/POPG/STG vesicles. All experiments were conducted in triplicate.

The pK_a_ values of the amino acids Arg (R) and Lys (K) are 12.48 and 10.53, respectively, indicating that the charge of the designed AMPs remains constant across a pH range of 5 to 8 (Figure 6A). This net positive charge enhances the peptide's ability to interact with microbial membranes. The virulence of *P. syringae* is associated with its entry through wounds or stomata and subsequent multiplication in the acidic apoplast, which is maintained by auxin‐induced proton pumps (Tsai and Schmidt 2021; Tossi et al. 1994). To evaluate the activity of these peptides against a Gram‐negative bacterial outer membrane model mimic composed of 7:3 POPE/POPG, we employed a fluorescence‐based calcein dye leakage assay (Domadia et al. 2010) at pH levels ranging from 5.4 to 8.0. Both VR18 and KG18 were found to interact with the membrane mimics, resulting in an increase in calcein fluorescence intensity by 80% and 75%, respectively (Figure 6B). In contrast, these peptides were unable to cause any such leakage in the case of a plant membrane model mimic system consisting of 5:4:3 POPC/POPE/STG (Figure 6B). Furthermore, both VR18 and KG18 demonstrated concentration‐dependent dye leakage in a bacterial inner membrane model mimic (3:1 POPE/POPG) at physiological pH (Figure S12A). To further assess the specificity of these peptides, we tested two well‐established AMPs, Polymyxin B and Melittin, for their interaction with a plant model membrane mimic. Our results indicated that both Polymyxin B and Melittin caused significant perturbation of the model membrane when compared to KG18 and VR18 (Figure S12B). Collectively, these findings hinted towards the specific selectivity of VR18 and KG18 towards bacterial model membranes.

Next, to assess the binding capacity of VR18 and KG18 to various membrane mimic models, we incubated the peptides VR18 and KG18 with LPS bicelles, as well as with model membranes composed of 7:3 POPE/POPG, 3:1 POPE/POPG, and 5:4:3 POPC/POPE/STG independently. A pulldown assay (Phan et al. 2015) followed by SDS‐PAGE analysis (Schägger 2006) revealed that more than 50% of both VR18 and KG18 preferentially bound to LPS bicelles and the bacterial membrane model liposomes. The ratio of bound and unbound fractions of the peptides with LPS bicelles and the bacterial membrane model liposomes was approximately 1:1, indicating a rapid interaction; however, a significant fraction of peptides was also detected in the unbound fraction. Nonetheless, the ratio of bound to unbound fraction for both peptides VR18 and KG18 with the plant membrane mimic 5:4:3 POPC/POPE/STG liposomes was approximately 1:3 for VR18 and 1:3.5 for KG18, indicating a majority of peptides remained unbound in this model (Figure 6C).

To further explore the selective membrane perturbation of the AMPs, we conducted ^31^P solid‐state NMR spectroscopy along with spectral shape analysis using bacterial (7:3 POPE/POPG) and plant (5:4:3 POPC/POPE/STG) model membrane mimics (Szoka Jr and Papahadjopoulos 1978). Both model membranes displayed powder patterns with a parallel edge at ~30 ppm and a perpendicular edge near 15 ppm. Upon the addition of the peptides at a concentration of 1 and 3 mol%, we observed instantaneous changes in the bacterial outer membrane model mimic (Figure 6D). The alterations observed at the parallel and perpendicular edges indicated a disturbance in the lipid distribution's homogeneity, resulting from peptide‐lipid interactions. Similar changes were noted for the bacterial inner membrane model mimics (Figure S12C). In contrast, the addition of VR18 and KG18 to the plant membrane mimics 5:4:3 POPC/POPE/STG liposomes did not result in any spectral changes, indicating that liposome integrity was maintained (Figure 6D). These findings indicated the negatively charged microbial membrane perturbing capability of VR18 and KG18 and the stability provided by stigmasterol to the plant membrane model mimic.

### 2.13 Bioactive Conformations of VR18 and KG18

**FIGURE 7:**
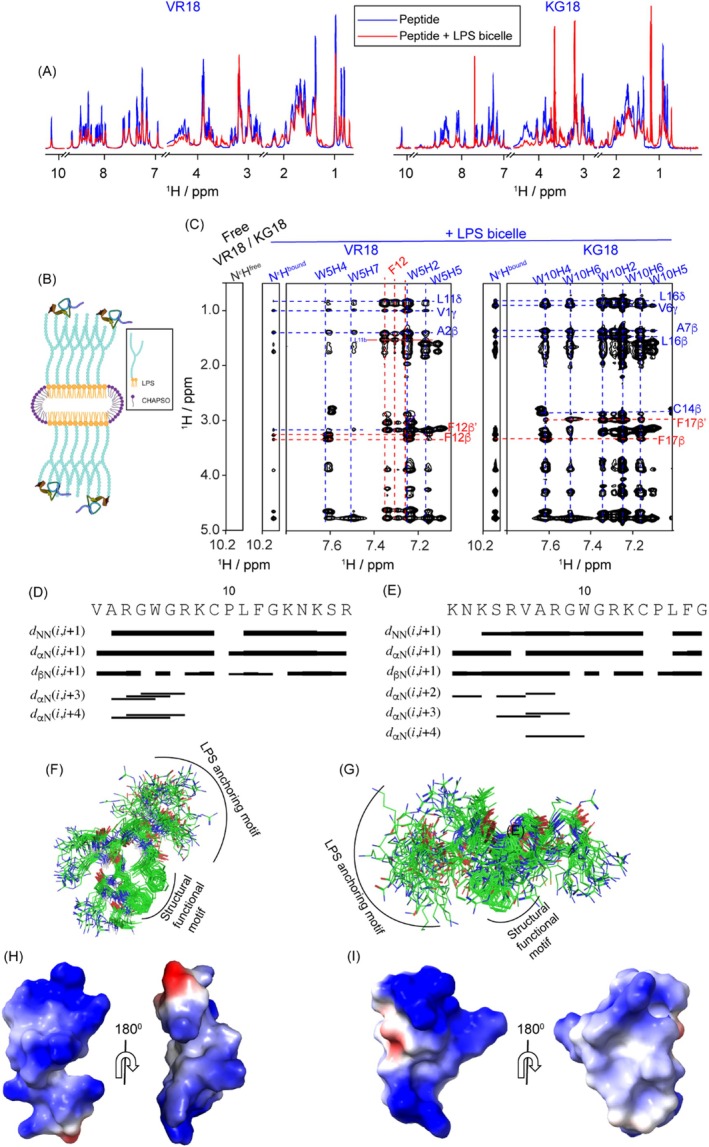
Solution‐NMR structural analysis of VR18 and KG18 in the presence of *Pseudomonas* LPS bicelles. (A) One‐dimensional ^1^H NMR spectra of VR18 and KG18, when titrated with *Pseudomonas* LPS bicelles, showed significant broadening of proton resonance and chemical shift changes, indicating interaction between the peptides and LPS. (B) Illustration of peptide interactions with LPS bicelles created using BioRender.com. Bicelles, as a bilayer, provide a native membrane environment for peptides to adopt secondary conformations. (C) 2D ^1^H‐^1^H *tr*NOESY spectra displayed medium and long‐range NOE connectivities for the LPS‐bound forms of VR18 (BMRB code: 36724) and KG18 (BMRB code: 36725) compared to their free forms. The LPS‐bound peptides showed numerous NOE cross‐peaks, indicating a well‐defined tertiary structure, whereas the free form lacked NOEs, suggesting an unstructured dynamic state. (D, E) Bar diagrams depicting sequential and medium‐range NOEs of VR18 and KG18 in the presence of Pseudomonas LPS bicelles. (F, G) Twenty lowest energy ensemble structures of LPS‐bound VR18 (RMSD = 1.06) (PDB accession code: 9LIB) and KG18 displayed a rigid structure (RMSD = 1.31) (PDB accession code: 9LJD) with tight backbone packing, showing the orientation of the LPS anchoring and structural‐functional motifs. (H, I) Electrostatic surface potentials of VR18‐bound and KG18‐bound LPS at a 180° angle demonstrated charge delineation, generated using MOLMOL software.

We used solution‐state NMR spectroscopy to investigate the 3D structures of VR18 and KG18 in the presence of *P. aeruginosa* LPS bicelles. Micelles and bicelles provide an optimal environment for high‐resolution NMR, as bicelles form bilayers that allow peptides to adopt native‐like conformations (Marcotte and Auger 2005). Upon the addition of 2 μL of LPS bicelles at pH 4.0, one‐dimensional proton NMR spectra of both VR18 and KG18 exhibited significant line broadening (Figure 7A). This condition is favourable for *tr*NOESY experiments, allowing for the determination of the 3D structure of peptides in the presence of LPS (Figure 7B), as they undergo fast to intermediate exchange with the bound macromolecule on the NMR time scale. The free peptide exhibited only certain intra‐αN (i,i) or sequential αN (i, i + 1) NOEs, suggesting a highly dynamic nature. However, in the presence of bicelles, strong αN (i, i + 1) and HN/HN NOEs were observed in most residues of KG18 and all residues of VR18 (except P10), indicating a transition from random coil to a well‐defined folded conformation (Figure 7C). Spectra analyses of LPS‐bound VR18 and KG18 also revealed several medium (i, i + 2/i + 3/i + 4), side chain/side chain, and long‐range (i to ≥ i + 5) NOEs (Figure 7D,E). Strikingly, in the case of VR18, the indole ring proton (N^ε^H) of W5, resonating at 10.17 ppm, showed contact with the aliphatic protons of V1, A2, L11 and F12. Several protons participated in establishing important long‐range NOE contacts with W5H4/W5H7/W5H2/W5H5, like L11CδH, F12βH, etc. (Figure 7C and Figure S13A). Side chain/side chain medium‐range NOEs were noted between V1/W5, A2/W5, and P10/F12. The N^ε^H of W10 of KG18 showed contact with the aliphatic protons of V6, A7, C14, L16, and F17 (Figure 7C and Figure S13A). The protons involved in long‐range NOE contacts with W10H4/W10H6/W10H2/W10H5 were L16CβH, L16CδH, F17βH, etc. Side chain/side chain medium‐range NOEs were noted between V6/W10, A7/W10, and W10/C14. Interestingly, the Cys‐Pro bond of VR18 or KG18 in LPS adopted trans conformation due to the presence of C9CαH/P10CδH and C14CαH/P15CδH NOE, respectively. The secondary chemical shift plot of VR18 and KG18 indicated minor deviations from the chemical shifts of random coil conformation for all the residues, indicating the adaptation of the peptides to secondary structures (Figure S13B). Absence of CαH/CαH as well as long‐range NH/NH contacts ruled out the presence of antiparallel β‐sheet conformation.

Using NOE‐based distance constraints, we determined 20 ensemble structures of LPS‐bound VR18 and KG18 with RMSD value of 1.06 and 1.31, respectively. 3D solution structure revealed that VR18 and KG18 had an amphipathic arrangement with a clear segregation of the polar residues and the hydrophobic residues in LPS containing environment. However, despite having equal number of positive residues, the orientation of the positively charged residues differed significantly in both peptides. In case of VR18, all the charged residues faced towards the solvent, but the ‘KNKSR’ motif was aligned in one‐ direction, whereas the structural‐ functional motif faced away from the KNKSR region. The R and K residues distinctly oriented themselves away from the hydrophobic cluster, forming a charged surface region. W5 was sandwiched between V1 and A2 on one end and F12 and L11 on the other. An association of the hydrophobic residues comprising of the WLF moiety formed a triad generating a loop‐like structure (Figure 7F). Of note, in our previous studies, LPS‐ bound VR18 adopted an amphipathic structure even at pH 7.4 (PDB ID: 7VQH) (Mohid, Sharma, et al. 2022). Overlaying the structures at pH 4.0 and 7.4 revealed a high RMSD value of 3.20, attributed to the dispersion of positively charged residues and strong electrostatic potential. At pH 7.4, residues V1, A2, W5, F12, and L1 formed a linear hydrophobic hub, while acidic pH enhanced hydrophobic packing, creating a more compact hub. (Figure S14).

We observed that the LPS bound KG18 occupies a larger surface area compared to VR18. This difference can be attributed to the C‐terminus of KG18, which lacks positive charges, leading to a more uniform distribution of charged residues. In contrast to VR18, KG18 does not exhibit segregation in the orientation of the KNKSR motif and structural‐functional regions. Additionally, we noted a compact hydrophobic core formed by the “WLF” moieties in KG18 (Figure 7G). The distances measured between the hydrophilic head groups—the ammonium (H_3_N^+^‐) group of lysine and the guanidinium group of arginine—ranged from 11 to 17 Å or were less than 10 Å, suggesting probable electrostatic interactions between the polar residues of the peptides and the phosphate head groups of the Lipid A component of LPS (Figure S15). These findings lead to the conclusion that the KNKSR motif plays a critical role in anchoring the peptides to LPS, followed by interactions between the hydrophobic WLF residues and the acyl chains, ultimately resulting in membrane perturbation. The electrostatic potential maps of LPS‐bound VR18 and KG18 further illustrate distinct surface charge distributions, underscoring the amphipathic nature and compact bioactive conformations of both peptides (Figure 7H,I).

### 2.14 Probing Peptide‐LPS Interactions Through MD Simulation

**FIGURE 8:**
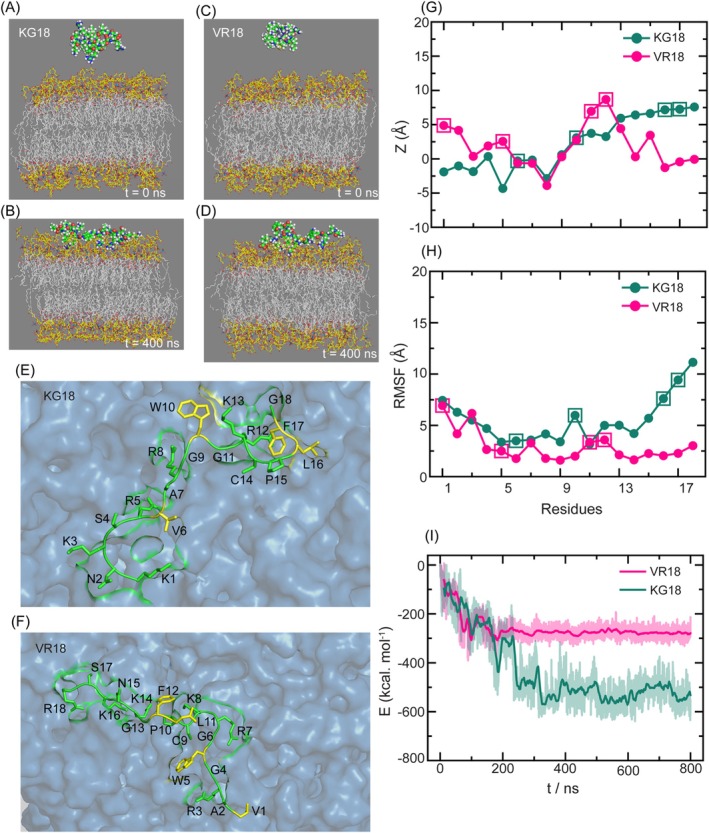
MD Simulations probing interaction of VR18 and KG18 with Re‐LPS. (A–D) The prepared peptide‐ReLPS lipid bilayer systems and their bilayer‐bound structures for KG18 and VR18 are shown side by side. The peptides are represented in ball‐and‐stick format, while the ReLPS lipids are depicted in licorice format, with tail groups coloured grey and head groups coloured yellow. (E, F) Close‐up views of the peptide‐bound conformations from MD simulations for KG18 and VR18 are shown. Residues marked in yellow indicate those that have been experimentally confirmed to interact with the bilayer surface, highlighting key regions of interaction. (G) The average positions of the amino acid center of masses relative to the lipid bilayer surface along the *z*‐axis are displayed. This analysis provides insight into how the peptides penetrate or associate with the membrane. (H) Root mean squared fluctuations (RMSF) for each peptide in their bilayer‐bound conformations are shown. Square‐marked points in plots G and H represent the experimentally observed residues that primarily interact with the bilayer surface, indicating their stability and role in membrane anchoring. (I) Coulomb interaction energy between the LPS anchoring motif within the peptides and the ReLPS bilayer is included.

We have used Molecular Dynamics (Mith et al. 2015) simulations to gain insights into the molecular structure, interactions, and dynamics of the peptides and a model ReLPS lipid bilayer. The peptide‐bilayer systems and the representative bilayer‐bound conformations for both peptides are shown in Figure 8A–D. A close view of representative peptide‐bound conformations obtained from simulation and the residues interacting with the bilayer found in the NMR studies are shown in Figure 8E,F.

To characterise the residue‐wise penetration depth of the amino acids, we have computed the average relative positions of all the amino acid residues (center of mass) for both the peptides in their bilayer‐bound conformations in reference to the bilayer surface along the *z*‐axis (Figure 8G). The Z‐coordinate for the bilayer surface was taken as 75 Å, which corresponds to the extent of the density profile of the Kdo sugar head groups (Figure S16). The results indicate that the extent of penetration varies near the termini regions, and the interacting residues obtained from MD simulations corroborate with the experimental findings.

We observed that the LPS‐binding motif for both peptides and the positively charged and hydrophobic residues like A7, R8, G9, G11, and R12 (KG18) and R3, G6, R7, K8, C9, and P10 (VR18) maintain close contact (within 5 Å in Figure 8G) with the bilayer surface. We also noticed that the C‐terminal of VR18 remains closer to the bilayer surface than the C‐terminal of KG18. The electrostatic repulsion between the C‐terminal COO^−^ group of KG18 and the negatively charged phosphate head groups causes this instability, whereas for VR18, the presence of the LPS‐anchoring motif helps the C‐terminal to maintain close contact with the bilayer surface. The residue‐wise root mean squared fluctuations (RMSF) for both peptides highlight the differences in flexibility in the bilayer‐bound state (Figure 8H).

The Coulomb interaction energy between the LPS‐anchoring motif (KNKSR, charge +3e) of KG18 and ReLPS lipid bilayer is much lower than that of the VR18 peptide (Figure 8I). This represented a stronger electrostatic interaction between the KG18 and the lipid bilayer in comparison to VR18, again corroborating well with solution‐state NMR findings. Due to the presence of the additional NH_3_^+^ group at the N‐terminal of KG18, the net positive charge of the KNKSR motif increases at neutral pH. Whereas, in the case of VR18, this motif forms the C‐terminal part, which results in a decrease in the net positive charge of this motif due to the presence of the additional COO^−^ group. As a result, the LPS binding motif present in the KG18 peptide shows a stronger electrostatic interaction with the negatively charged phosphate lipid head groups than the VR18 peptide. Both peptides remain bound to the bilayer surface, but with significant differences in structure, binding mode, and interactions.

We have further analysed the extent of perturbation of bilayer structure on peptide binding. We find that in the KG18‐bound conformations, the density of the Kdo sugar head groups on the peptide‐interacting bilayer leaflet is much lower than for the VR18 peptide (Figure S17). This observation further highlights that KG18 has stronger membrane disruption abilities as compared to VR18.

## 3 Discussion

Plant disease control has traditionally relied on methods such as chemical pesticides, resistant cultivars, and antimicrobials. While these strategies have proven effective in some contexts, they come with significant drawbacks. The excessive overuse of chemical pesticides not only leads to environmental pollution but also contributes to the emergence of resistant bacterial strains, highlighting the urgent need for alternative solutions. Recent advancements in genetic modifications offer promising strategies for broad‐spectrum disease resistance. This can be achieved by either upregulating genes involved in systemic acquired resistance or manipulating plant defence signalling pathways (Andolfo et al. 2016). Recombinant DNA techniques have previously been employed to regulate plant hormones to enhance plant disease resistance. However, modifications to critical pathways such as salicylic acid biosynthesis and ethylene/jasmonic acid signalling have often led to trade‐offs. These trade‐offs can include reduced plant growth and compromised fitness (Gray 2004; Mauch‐Mani et al. 2017).

The application of antimicrobial peptides (AMPs) in plants represents a novel and promising strategy for combating phytopathogens and enhancing disease resistance. For instance, Osusky et al. (1999) demonstrated that the expression of a cecropin‐melittin cationic peptide chimera in *Solanum tuberosum* L. conferred resistance to both fungal and bacterial phytopathogens (Osusky et al. 1999). Similarly, Imamura et al. (2010) reported that the expression of thanatin in *Oryza sativa* improved the plant's tolerance to rice blast disease (Imamura et al. 2010). Despite these advancements, there remains a significant gap in our understanding of the mechanistic pathways through which AMPs operate in planta and their interactions with the host defence system and metabolic processes. Our study addresses this gap by exploring two rationally designed chimeric peptides, VR18 and KG18, which exhibit enhanced antibacterial activity and favourable biophysical properties compared to their precursor peptides. By elucidating the mechanisms of action of these AMPs, we aim to advance the development of effective strategies for plant disease management.

In vitro analyses revealed that VR18 and KG18 peptides possess broad‐spectrum antibacterial activity against various phytopathogens, including *Pseudomonas syringae* , with enhanced efficacy compared to their precursor peptides. These peptides were found to be non‐cytotoxic, protease stable, and non‐allergenic, making them excellent candidates for transgenic applications. Microscopy studies confirmed that their primary mode of action involves bacterial membrane lysis, specifically through the perturbation of membrane surface integrity. Biophysical studies further elucidated their interaction with lipopolysaccharides (LPS) in the bacterial outer membrane, indicating a detergent‐like mechanism of action. Notably, VR18 and KG18 exhibited selective targeting of bacterial membranes without affecting plant membrane models, an essential characteristic for minimising off‐target effects in transgenic plants. This selective antimicrobial activity positions these peptides as promising tools for enhancing disease resistance in crops through genetic engineering.

The transgenic expression of peptides, VR18 and KG18 in *Nicotiana tabacum* significantly increases the resistance to *Pseudomonas syringae* , as evidenced by a delay in symptom onset and reduced disease severity. Importantly, the constitutive expression of these peptides did not adversely affect the plant's phenotype, growth, or metabolic processes. In contrast to wild‐type plants, which displayed severe chlorosis and stunted growth upon infection, the transgenic plants exhibited only mild symptoms and maintained normal development. In our analysis, we found that the VR18 encoding gene was expressed a little more in line 1, while line 2 showed a slight increase in gene expression of KG18. Despite these differences, the consistent production of both peptides in all the lines was enough to stop the growth of *P. syringae* after infection and to reduce disease symptoms. Overall, the expression of the VR18 encoding gene was more than that of the KG18 expressing gene. Moreover, the VR18 and KG18 expressions did not activate the plant's endogenous defence mechanism, even during pathogen attacks, suggesting that these peptides operate independently of the host immune pathways. This unique feature may mitigate risks associated with unintended stress responses in genetically modified plants. It is likely that the pathogenic invaders are impeded by the host‐derived VR18/KG18 peptides, which may disrupt the pathogen's membranes and hinder their invasion.

MD simulations offered deeper insight into the mechanism of action of the peptides VR18 and KG18. The positively charged residues of these peptides interact electrostatically with the phosphate head groups of LPS, facilitating initial binding to the membrane. Subsequently, hydrophobic residues interact with the acyl chains of LPS, leading to membrane disruption. This dual interaction mechanism not only demonstrates the effectiveness of VR18 and KG18 in bacterial lysis but also suggests their potential for broad‐spectrum applications. Additionally, the peptides exhibited bioactivity across a wide pH range, a critical property considering the pH fluctuations that occur in plant apoplasts during pathogen infection.

Importantly, the use of AMPs such as VR18 and KG18 may also offer a valuable strategy for mitigating the rise of antimicrobial resistance. Unlike conventional antibiotics that often target specific bacterial pathways or enzymes, AMPs disrupt microbial membranes via biophysical interactions that are less prone to resistance development. The multi‐modal and non‐specific nature of membrane disruption, combined with rapid bactericidal action, reduces the likelihood of bacteria evolving stable resistance mechanisms. Furthermore, because AMPs are derived from natural host‐defence molecules, they are typically biodegradable and less likely to accumulate in the environment compared to synthetic agrochemicals. This biodegradability, along with their selective activity towards pathogens and minimal toxicity to plants, animals, and beneficial microbes, supports their application as eco‐friendly alternatives to chemical pesticides. Implementing AMP‐based strategies could therefore improve environmental safety by lowering pesticide load and minimising off‐target ecological damage, while also contributing to food safety by reducing the risk of resistant or persistent pathogens entering the agricultural supply chain.

Notably, the peptides, VR18 and KG18 confer a unique set of benefits compared to conventional pathogen‐resistant transgenic plants. Most existing strategies rely on altering the acquired defence system of the plants. In contrast, VR18 and KG18 act independently of the host immune system by directly targeting bacterial membranes, thereby avoiding such physiological costs. Their mechanism of action, via selective interaction with conserved lipopolysaccharide components, reduces the likelihood of resistance development and ensures pathogen specificity. Additionally, their protease stability, broad pH tolerance, and non‐cytotoxicity further support their implementation under variable environmental conditions. Importantly, constitutive expression of VR18 and KG18 transgenic plants represents a next‐generation approach in plant pathogen resistance; mechanistically distinct, ecologically safer, and more durable than many current strategies. These advantages make them strong candidates for sustainable disease management and food security solutions.

Despite these promising findings, several limitations warrant further investigation. For instance, while the peptides, VR18 and KG18, demonstrated efficacy against *P. syringae* , their effectiveness against a broader range of bacterial and fungal pathogens should be explored in vivo to confirm their potential for broad‐spectrum activity. Furthermore, although no adverse effects were observed on plant growth and metabolism in our study, long‐term field studies are essential to assess the ecological and evolutionary implications, including the risk of resistance development in target pathogens. Comparative analyses with existing AMPs and transgenic strategies would also provide valuable context for evaluating the relative advantages of VR18 and KG18. Furthermore, the effect of the constitutive expression of these peptides on the microbes in symbiotic association with the plants has to be assessed. Exploring alternative strategies, such as pathogen‐induced expression of the peptides, should also be considered. Lastly, implementing KG18 and VR18 transgene applications in food crops could significantly enhance not only crop health but also human health outcomes.

In conclusion, VR18 and KG18 transgenic plants represent a next‐generation approach in plant pathogen resistance: mechanistically distinct, ecologically safer, and more durable than many current strategies. These advantages make them strong candidates for sustainable disease management and food security solutions. With additional optimisation and validation, these peptides could help reduce reliance on chemical pesticides while contributing to increased crop yields and enhancing global food security.

## 4 Methods

### 4.1 Antibacterial Assay

*Pseudomonas syringae* pv. *tabaci* (procured from Indian type culture collection number), IARI, New Delhi was a humble gift from Prof. Nir Singha Dey, ILS, Orissa. *Pseudomonas syringae* pv. *tabaci* was grown in King's broth (20 g/L peptone, 10 mL/L glycerol, 1.5 g/L dipotassium hydrogen phosphate, 1.5 g/L magnesium sulfate) containing 50 μg/mL Rifampicin at 28°C overnight under shaking conditions. *Xanthomonas oryzae* pv. *oryzae* (ATCC BX043) was grown in Peptone Sucrose broth (1% peptone, 1% sucrose) at 28°C for 72 h under shaking conditions. *Xanthomonas campestris* pv. *campestris* was grown in Peptone Sucrose Potato broth (0.5% peptone, 2% sucrose, 200 g/L potato starch) at 28°C for 48 h under shaking conditions. *Xanthomonas campestris* pv. *vesicatoria* was grown in Nutrient Broth at 28°C for 48 h under shaking conditions. *Pseudomonas aeruginosa* was grown in King's broth at 37°C overnight under shaking conditions. *Enterococcus faecalis* , *Staphylococcus aureus* , *Klebsiella pneumoniae* , *Acinetobacter baumanii* and *Escherichia coli* were grown in Luria broth at 37°C overnight under shaking conditions. All bacterial media were purchased from Himedia Laboratories Pvt. Ltd., Mumbai, India.

Actively growing bacterial cultures (mid‐logarithmic) were pelleted at 5500 rpm for 10 min and washed with 10 mM phosphate buffer (pH 7.4) thrice and resuspended in the same to obtain a cell suspension containing 10^6^ CFU/mL. Modified microbroth dilution assays were performed, where the individual cell suspensions containing 10^5^ CFU/mL were supplemented with varying concentrations (ranging from 1 μM to 100 μM) of the designed peptides, VR18 and KG18 (GenScript, USA), separately. Negative controls comprising cells only and positive controls containing cells treated with Polymyxin B were also maintained simultaneously. After incubation at appropriate growth conditions and culture medium mentioned above, specific for each bacterial strain, OD_630_ was recorded to monitor cell growth in control and treated samples. Normalisation was done using the positive control to determine the percentage of bacterial growth as well as the percentage of bacterial growth inhibition. The concentration of peptide at which the percentage reduction in the number of viable cells is ≥ 99% served as the Minimal Inhibitory Concentration (MIC_99%_). All the experiments were performed in triplicate.

### 4.2 Membrane Permeabilization Assay

*Pseudomonas syringae* pv. *tabaci* cells were prepared and incubated with Propidium Iodide. The increase in fluorescence intensity was checked upon the addition of peptides. For details, please see section 1.1 of Supporting Information.

### 4.3 Cell Viability Assay

The cell viability of HEK293 cells in the presence of VR18 and KG18 was monitored by MTT assay following the standard protocol. For details, please see section 1.2 of Supporting Information.

### 4.4 Plant Extract Stability Assay

The stability of VR18 and KG18 in the presence of plant proteases present in plant leaf extract was monitored using a modified version of a previously reported protocol (Li et al. 2002). Briefly, 1 g of 1‐month‐old tobacco leaf ( *Nicotiana tabacum* ) was crushed in liquid nitrogen, followed by extraction in 10 mM phosphate buffer (pH 7.4). The suspension was pelleted at 13500× g for 5 min, and the supernatant was collected. 1 mg of the respective peptide was added to a stock of 20% v/v plant extract in 10 mM phosphate buffer (pH 7.4) and incubated at 37°C. 100 μL of aliquots were withdrawn at appropriate time intervals, and the reaction was stopped using 1% trifluoroacetic acid (TFA). Plant extract (20% v/v) was used as a negative control. The aliquots were subjected to a LC‐20AT reverse phase HPLC system (SHIMADZU, Japan) equipped with a Phenomenix C_18_ column (250 × 10 mm, pore size 100 Å, particle size 5 μm) at room temperature, using acetonitrile and water with 0.1% TFA as the solvents. A flow rate of 1 mL/min was maintained. The percentage of peptide remaining in the plant extract at different time intervals was analysed by peak integration using SPINCHROME CFR software, and the area obtained was normalised with respect to the free peptide without the plant extract.

### 4.5 Molecular Dynamics Simulation

A symmetric *E. coli* ReLPS (Brandner et al. 2024) lipid bilayer was constructed using the *Bilayer Builder* in CHARMM‐GUI (Jo et al. 2008; Lee et al. 2016, 2019; Wu et al. 2014) and parameterised using CHARMM36m (Huang et al. 2017) force field. VR18 and KG18 peptides were modelled using CHARMM36m force field, and one peptide of each type was placed around 1 nm distance on the top of the converged and equilibrated ReLPS bilayer structure. Simulations were performed using GROMACS (Abraham et al. 2015) v2021.5 and v2024.3 under periodic boundary conditions. Long‐range electrostatic interactions were handled using the particle mesh Ewald (PME) summation method (Essmann et al. 1995). Bonds containing hydrogen atoms were constrained using the LINCS algorithm (Supporting Information, Section 1.16).

### 4.6 Generation of VR18/KG18 Expressing Transgenic Plants

Agrobacterium‐mediated transformation of tobacco leaf explants was carried out using standard methodology. Briefly, explants were collected from 10‐day‐old healthy seedlings grown in solid Murashige‐ Skoog medium (MS salts and 3% sucrose) under aseptic conditions. *Agrobacterium tumefaciens* LBA4404 strain harbouring pCAMBIA1304 vector with VR18 and KG18 construct was used to transform tobacco explants. *Agrobacterium* was grown in Luria Broth (Horvath et al.) media with kanamycin (50 mg/L) and rifampicin (50 mg/L) at 28°C under continuous shaking for 48 h. Cells were then harvested by centrifugation at 6000 rpm, 4°C, resuspended in MS medium supplemented with acetosyringone (100 μM), and adjusted to a final OD_600nm_ of 0.8. Leaves were excised of tip and petiole and incubated in MS medium supplemented with zeatin (1 mg/mL) and 1‐Naphthaleneacetic acid (0.1 mg/mL) for 2 days at 25°C under 16/8 h light–dark cycles. Explants were then immersed in *Agrobacteria* suspension for 5 min, shaking occasionally, and returned to the same media after removal of excess culture liquid. Following 2 days of co‐incubation at 25°C under 16/8 h light –dark cycles, the explants were transferred to regeneration media (MS medium, 1 mg/L zeatin, 0.1 mg/mL 1‐Naphthaleneacetic acid, 250 mg/L Cefotaxime, and 75 mg/L hygromycin) and incubated till callus induction. Calli were maintained on 75 mg/L hygromycin‐containing media until shoots developed. Emerged shoots were then detached from the callus and transferred to a rooting media (MS medium, 250 mg/L cefotaxime, and 75 mg/L hygromycin) in jam bottles. Plantlets with profuse roots were selected and transferred to soilrite, kept under moist conditions in the culture room for hardening. Finally, transgenic plants were planted in larger pots and maintained in glasshouses for seed production. T_1_ seeds obtained from transgenic T_0_ plants were surface sterilised with 70% ethanol, followed by repeated washing in autoclaved water. Then, the seeds were allowed to germinate on MS media supplemented with 75 mg/L hygromycin. The germination rate of 90%–95% was routinely obtained in T_1_ and T_2_ generation seeds, and all the experiments were performed with T_2_ generation plants. Vector control plants transformed with *Agrobacterium* harbouring only pCAMBIA1304 vector without the peptide construct were also generated in a similar way.

### 4.7 Real Time PCR Analysis to Check the Expression of VR18 and KG18 Constructs in Transgenic Plants

Total RNA was isolated from vector control and transgenic tobacco leaves using RNAiso Plus (TaKaRa Bio) following the manufacturer's protocol. RNA concentration was measured using the Thermo Scientific ND‐1000 nano‐drop spectrophotometer. cDNA was synthesised from 5 μg of high‐quality total RNA employing 5 μM random hexamer primer, 10 U Thermo Scientific Revert Aid Reverse Transcriptase, 1mMdNTPs and 0.5 U Thermo Scientific RiboLock RNase Inhibitor in 1X RT buffer using the manufacturer's protocol. 2.5%–5% of the cDNA reaction mix was used as a template in a 20 μL reaction containing PowerUp SYBR Green Master Mix (Applied Biosystems) and gene‐specific primers (0.15 μM). Quant Studio Real‐Time PCR System (Applied Biosystems) was used to perform PCR and analysis. For each cDNA, 3 technical replicates were used. Amplification of the specific product was confirmed by obtaining a single peak in melt curve analysis, the first derivative of fluorescence (dF/dT) v/s temperature plot, and 2.5% agarose gel electrophoresis of the PCR end‐products. The expression of *EF1*α was checked for sample integrity. All experiments were repeated with three independent RNA preparations.

### 4.8 Infection of Transgenic Plants with *Pseudomonas syringae* pv. Tabaci

*Pseudomonas syringae* pv. *tabaci* was cultured in King's Broth medium containing rifampicin (50 μg/mL) at 28°C under continuous shaking conditions till the OD_600nm_ reached 0.6. Next, the cells were pelleted and resuspended in sterile water containing 100 μM acetosyringone, to a final OD_600nm_ of 0.05. The bacterial suspension was syringe infiltrated on the ventral surface of the leaves of 45‐day‐old transgenic tobacco plants, transgenically expressing the VR18 and KG18 constructs. Wild‐type and vector control tobacco lines were also simultaneously infected with the same bacterial suspension. All plants were maintained at a humidity of 85%–90% till the onset of visible symptoms, mostly until after ~72 h (Deb et al. 2020).

The pathogenicity of *Pseudomonas syringae* in vector control plants and transgenic plants expressing VR18 and KG18 was assessed using the leaf filtration assay mentioned previously, followed by crushing the infected leaf samples after 72 h of infection to prepare a suspension. The suspension was centrifuged at 6000 rpm, and 100 μL of the supernatant was spread onto King's agar medium composed of 50 μg/mL rifampicin. The CFU from both vector control and transgenic plants was determined, and the percent reduction in CFU of transgenic lines was calculated with respect to the vector control plants (Deb et al. 2020).

### 4.9 Determination of Generation of Reactive Oxygen Species

The detection of superoxide radicals was performed using Nitro blue tetrazolium (NBT) staining. Briefly, tobacco leaves, uninfected and infected, from control and transgenic plants were vacuum infiltrated in an NBT staining solution (0.1% NBT, 10 mM sodium azide, 50 mM potassium phosphate buffer at pH 7.0–7.4) for 1 h. Next, the leaves were incubated in the staining solution for an additional 1–2 h, followed by dechlorophylisation in a bleaching solution containing ethanol, acetic acid, and glycerol (3:1:1, v/v/v) under boiling conditions. The stained leaves were then visualised and photographed under white light. Accumulation of hydrogen peroxide in externally infected leaves, control and, transgenic, was detected using 3,3′‐diaminobenzidine (DAB) staining. Firstly, the leaves were excised from the petioles and immersed in DAB staining solution (1 mg/mL DAB in 10 mM phosphate buffer) and left overnight at 28°C under shaking conditions in the dark. The next day, the leaves were put in a bleaching solution of ethanol: acetic acid: glycerol (3:1:1) followed by washing in boiling water to remove the chlorophyll. The leaves were visualised and photographed under white light (Basak et al. 2024).

### 4.10 Detection of Abiotic and Biotic Stress in Transgenic Plants Upon Pathogen Attack

Total RNA was isolated from the uninfected as well as infected vector control and transgenic tobacco leaves, and cDNA was synthesised as described above. 2.5%–5% of the cDNA reaction mix was used as a template in a 20 μL reaction containing PowerUp SYBR Green Master Mix (Applied Biosystems) and gene‐specific primers (0.15 μM). Quant Studio Real‐Time PCR System (Applied Biosystems) was used to perform PCR and analysis. Data was normalised based on the expression of the housekeeping *EF1*α gene transcripts. Amplification of specific products was confirmed by obtaining a single peak in melt curve analysis, the first derivative of fluorescence (dF/dT) v/s temperature plot. Relative expression levels of transcripts were determined using the 2^−ΔΔcT^ method. All experiments were repeated with three independent RNA preparations.

### 4.11 LC‐ MS/MS to Determine Plant Metabolites

500 mg of leaves from wild‐type and transgenic plants were excised and crushed into powder after freeze‐drying in liquid nitrogen. Methanol was added to the homogenates and kept at 4°C overnight. After 10 min of centrifugation (4800× g), at room temperature, the supernatant was transferred into a microtube and filtered through Sep‐Pak C18 cartridge (Waters). The cartridge was washed with a methanol gradient (20%–30%), and the elution was done with 100% methanol. The eluted extract was fractionated in a 1.7 μm BEHC 18 analytical column equipped with an ACQUITY UPLC M‐Class before being passed on to XEVO G2‐XS QTof mass spectrometry (Water Corporation, Milford MA, Massachusetts, USA) in an LC–MS/MS positive mode system. A gradient combination of water and acetonitrile made up the liquid phase. Feature detection, metabolite identification, and peak alignment were performed using Progenesis QI with default parameters. The PlantCyc database was used to identify the metabolites with mass tolerance (±5 ppm) (Hawkins et al. 2021). The peak intensity data were first filtered by variance and median abundance value (5%) and then normalised by median and Pareto scaling for statistical analysis in MetaboAnalyst 6.0 (Pang et al. 2024). The metabolomics data have been deposited to MetaboLights (63) repository with the study identifier MTBLS12255.

### 4.12 Statistical Analysis

All biophysical assays were done in triplicate. Cell viability assay was also done in triplicate. For biological experiments, three independent lines expressing KG18 and VR18 were used. qRT‐PCR analyses were performed with three independent plants of the same age group, in three technical triplicate. All statistical analysis was performed in Graph Pad Prism 8. Results are expressed as mean ± SEM. Statistical significance was determined using the two‐tailed, unpaired Student's t‐test in column analysis. Two‐way ANOVA was used wherever essential. *p* ≤ 0.05 (95% confidence) was considered significant in all studies.

### 4.13 Other Experimental Studies

The detailed methods of saturation transfer double difference (STDD) NMR, scanning electron microscopy (SEM), atomic force microscopy (AFM), tryptophan fluorescence spectroscopy, isothermal titration calorimetry (ITC), dynamic light scattering (DLS), ^31^P solution state NMR, ^31^P solid‐state NMR calcein dye leakage, liposome and LPS bicelle pull‐down assay etc. can be found in Supporting Information linked to this article.

## Author Contributions

A.B. conceived, designed, analysed, overall supervised as well as funded the research work; K.B. performed the biophysical experiments, including NMR and all plant work; S.M. conducted MD simulation under the supervision of S.C. and R.B.; D.R. performed LC–MS metabolite data analysis with S.S.; S.R. did the MTT assay; Dibakar S did the NMR analysis of KG18 peptide; D.S. and D.K.L. performed ^31^P solid‐state NMR and its analysis; K.B. and A.R. did AFM experiments with Dulal S.; H.I. initiated the project and edited the manuscript; I.B. analysed the bacterial work and edited the manuscript; R.D. did the molecular biology work related to pCAMBIA cloning part; P.K. supervised the transgenic plant work and its analysis; A.B., K.B., P.K., I.B., S.M., D.R., S.R., Dibakar S. analysed the data; A.H. performed ML based ADMET analysis; K.B., H.I., I.B. and A.B. wrote the manuscript; all authors have given approval to the final version of the manuscript.

## Disclosure

*Notes*: The three‐dimensional structures of VR18 and KG18 bound to the LPS bicelles of *Pseudomonas aeruginosa* have been deposited in the Protein Data Bank (PDB). The PDB accession code of the structure of VR18 bound to LPS bicelles is 9LIB with a BMRB accession number is 36724, while the PDB accession code of the structure of KG18 bound to LPS bicelles is 9LJD and, the BMRB code is 36725. The raw metabolomics data have been deposited in EBI MetaboLights under the reference ID MTBLS12255 (https://www.ebi.ac.uk/metabolights/MTBLS12255).

## Conflicts of Interest

The authors declare no conflicts of interest.

## Supporting information


Data S1.

Table S4.

Table S5.

Table S6.

## Data Availability

All data needed to evaluate the conclusions in the paper are present in the manuscript and/or the Supporting Information.
