# Supplementary material for: Transgenic Tobacco Plants Expressing Synthetic Peptides: A Functional and Structural Analysis for Pathogen Resistance
Source: Plant Biotechnol J. 2025 Sep 15;24(2):526–46. doi: 10.1111/pbi.70287 (PMC12906832; doi:10.1111/pbi.70287)
Supplement: Supplementary file 1 — Data S1. [file PBI-24-526-s001.pdf]

# Supporting Information

## Transgenic tobacco plants expressing synthetic peptides: A functional and structural analysis for pathogen resistance

Karishma Biswas,[1,2] Sudipta Mitra,[3] Dibakar Roy,[4] Sanhita Roy,[2] Dibakar Sarkar,[1,2] DeokHyun Son,[5] Rohit Das,[4] Anuradha Roy,[6] Dulal Senapati,[6] Humaira Ilyas,[1] A. Harikishore,[7] Ranjit Biswas,[3] Suman Chakrabarty,[3] DongKuk Lee,[5] Indranil Biswas,[1,8] Sudipto Saha,[4] Pallob Kundu,[4] Anirban Bhunia[1,*]

[1]Department of Chemical Sciences, Unified Academic Campus, Bose Institute, Kolkata 700 091, India

[2]L V Prasad Eye Institute, Hyderabad- 500034, India

[3]Chemical, Biological and Macromolecular Sciences, S. N. Bose National Centre for Basic Sciences, Kolkata 700106, India

[4]Department of Biological Sciences, Unified Academic Campus, Bose Institute, Kolkata 700 091, India

[5]Department of Fine Chemistry, Seoul National University of Science and Technology, Seoul 01811, Korea

[6]Chemical Sciences Division, Saha Institute of Nuclear Physics, Kolkata 700 064, India

[7]School of Biological Sciences, Nanyang Technological University, 60 Nanyang Drive, Singapore 637551

[8]Department of Microbiology, University of Kansas Medical Center, Kansas City, Kansas, USA

*Address for correspondence

Anirban Bhunia. Email: bhunia@jcbose.ac.in

Running Title: *De novo* designed antimicrobial peptides against phytopathogen

## 1. *Experimental Procedures*

### *1.1. Membrane permeabilization assay*

*Pseudomonas syringae* pv. *tabaci* cells were grown till mid-logarithmic phase. The cells were pelleted, washed and resuspended in 10mM phosphate buffer (pH 7.4) as mentioned above to obtain a suspension containing $10^5$ CFU/ml. 10µM of Propidium Iodide (Sigma Aldrich Co., St. Louis, USA) was added to the cell suspension and incubated at room temperature, under shaking conditions for 30min. Using Hitachi F-7000 FL spectrophotometer (Tokyo, Japan), the fluorescence intensity was measured at 25 ˚C for 30 min at an excitation and emission wavelength of 535 nm and 617 nm respectively. After obtaining the fluorescence intensity of the stabilized cells, peptides were added at increasing concentrations, and the increase in fluorescence intensity was recorded. Normalization was done using measurement of cells treated with Polymyxin B that served as control. All the experiments were performed in triplicates.

### *1.2. Cell viability assay*

HEK293 cell viability was monitored by MTT assay. Briefly, the cells ($2x10^4$ cells/well) were seeded into 96 well plates, grown overnight and treated with different concentrations of VR18 or KG18 (up to 4x MIC) for 24 h. Control cells with no peptide treatment were also maintained simultaneously. Cell viability was then assessed by incubating the control and peptide treated cells using 100 µl of MTT (2mg/ml) for 3 h. The formazan crystals formed were dissolved in dimethyl sulfoxide (DMSO) and absorbance at 570 nm was recorded using SpectraMax M3 (Softmax Pro 6.3) multiplate reader. All experiments were done in triplicates. Cell viability was calculated using the equation:

% cell viability = [$OD_{570}$ of experiment well/ $OD_{570}$ of control well] x 100.

### *1.3. Prediction of allergenicity of the peptides and toxicity assessment*

The prediction of allergenicity of VR18 and KG18 was done using a webserver AlgPred (http://www.imtech.res.in/raghava/algpred/) that uses the amino acid sequence of peptides or proteins to be studied. A readseq program, (http://iubio.bio.indiana.edu/soft/molbio/readseq/) is used by the server that reads the protein sequences in standard formats like EMBL, FASTA, GCG or plain text format (Saha & Raghava, 2006). SVM- based method using amino acid composition of the peptides were used to obtain comprehensive information like score, threshold, positive predictive value (PPV), negative predictive value (NPV) and distance from threshold of the prediction. Any value of PPV >80% is indicative of the protein being an allergen.

The toxicity assessment of the peptides were done by determining the ADMET properties of VR18 and KG18 using the ADMET-AI server (https://admet.ai.greenstonebio.com) (Swanson *et al.*, 2024).

### *1.4. Saturation Transfer Double Difference (STDD) NMR*

Bruker Avance III 500 MHz equipped with a 5 mm SMART probe was used for STDD experiments. Peptide stocks (1mg/ml) and *P. syringae* cell suspension ($OD_{600nm}$) were prepared in 100% deuterated phosphate buffer (pH 4.5). A standard STD pulse program was used with saturation frequency at -1 ppm and an off- resonance of 40 ppm. The on- resonance saturation frequency selectively saturated the cells, while the off- resonance frequency did not resonate either the cells or the peptides. Control experiments with only peptides and only cells were done to generate respective spectra. Selective irradiation of cells was achieved by a series of 40 Gaussian- shaped pulses, each of 49 ms and separated by 1 ms delay between pulses. A saturation time of 2s was employed. Subtraction of the off- resonance spectra from the on- resonance spectra yielded the STDD spectrum. A total of 2048 and 1024 scans were performed for the STD and reference spectra respectively,

### *1.5. Scanning Electron Microscopy*

*P. syringae* cells were grown to mid-logarithmic phase, pelleted, washed and resuspended in 10mM phosphate buffer (pH 7.4) to obtain a suspension containing $10^5$ CFU/ml. The cell suspensions were incubated with VR18 and KG18 at concentrations of 0.5x MIC, 1x MIC and 2x MIC for 1h at 28˚C. Untreated cells served as control. 10 µl of the untreated and treated cells were spotted on clean glass coverslips, washed and fixed with 4 % p- formaldehyde for 1 h at 4 ˚C. Following fixation, the coverslips were washed twice and dehydrated by a graded series of ethanol (30 %, 50 %, 70 %, 80 %) and air dried overnight. The coverslips were then subjected to coating with gold palladium for 75s under high vacuum evaporator. Scanning electron microscope (Carl Zeiss- Model EVO 18, Carl Zeiss, Germany) was used to visualize at a magnification of 5000x.

### *1.6. Atomic Force Microscopy*

Mid- logarithmic phase grown *P. syringae* in King's broth supplemented with 50µg/ml Rifampicin was centrifuged, washed thrice with 10mM phosphate buffer (pH 7.4) and resuspended in the same. Cell suspension consisting of $10^5$ CFU/ml was treated with concentrations of 1x MIC and 2x MIC of VR18 and KG18 for 30 mins. Untreated cells were used as control. 10 µl of sample was spotted on a thin layer of mica sheet (SPI Supplies, West Chester, USA) attached on a glass slide. The sample was air dried, followed by further drying under argon atmosphere. AFM Bruker, Biocatalyst, equipped with silicon nitride probe SNL-10 (Bruker) was used for acquiring solid-

phase AFM images. The AFM software suite installed in the AFM instrument was used for data acquisition and analysis.

### *1.7. Tryptophan fluorescence spectroscopy*

The intrinsic fluorescence of tryptophan of VR18 (W5) and KG18 (W10) in 10mM phosphate buffer (pH 7.4) in absence and presence of *P. aeruginosa* serotype 10 LPS (Sigma Aldrich Co., St. Louis, USA) micelles and LPS bicelles were recorded using Hitachi F-7000 FL spectrophotometer (Tokyo, Japan). LPS micelles were prepared by dissolving 10 mg of LPS in 10 mM phosphate buffer (pH 7.4). LPS bicelles were prepared by dissolving 2 mg of LPS and 1.5 mg of CHAPSO (Avanti Polar Lipids, Alabaster, AL) in 10 mM phosphate buffer (pH 7.4) containing 150 mM NaCl maintaining the lipid/detergent ratio (q- ratio) between 0.25-0.3 and allowed to hydrate for 3 h. After hydration, the detergent containing LPS was subjected to five freeze and thaw cycles. The fluorescence spectra of 10 μM peptide were measured upon addition of increasing concentration of LPS (10 to 50 μM) at a spectral range of 300- 400 nm using excitation wavelength of 280 nm, slit width of 5 nm, in quartz cuvette of path length 0.1 cm.

Steady- state anisotropy was measured using QM-400 L-format fluorimeter (HORIBA, Canada PTI) equipped with polarized accessories. The fluorescence anisotropy (r) values were calculated using:

$$r = (I_{VV} - G \times I_{VH}) / (I_{VV} + 2 \times G \times I_{VH}) \quad (1)$$

where $I_{VV}$ and $I_{VH}$ are the vertically and horizontally polarized components with excitation by vertically polarized light at 280 nm. G denotes the sensitivity factor of the instrument.

### *1.8. Isothermal titration calorimetry*

The thermodynamics of the interaction of VR18 and KG18 with LPS was determined using TA-affinity ITC (TA instruments, Lukens Drive, New Castle, DE, USA). All peptides and LPS was dissolved in 10mM phosphate buffer (pH 4.5), filtered and degassed. 0.1 mg/ml of LPS was loaded in the sample cell to a volume of 182 μl and was titrated with 1 mg/ml of VR18 or KG18; loaded in the syringe. A total of 25 injections were performed, with each injection containing 2 μl of peptide at an interval of 2 min, at 25 ˚C. Analysis of raw data was done using NanoAnalyze 3.7.5 software provided with the instrument. Fitting of each plot was done employing an independent binding site model to determine the number of binding sites (n), dissociation constant ($K_D$), change in enthalpy (ΔH), free energy of binding (ΔG) and entropy (ΔS). Gibbs free energy for both reactions was evaluated using the equations:

$\Delta G = -RT \ln K_A$ and $\Delta G = \Delta H - T\Delta S$.

### *1.9. Dynamic light scattering*

A Malvern Zetasizer Nano S (Malvern Instruments, UK) equipped with a 4mW He-Ne laser (633 nm) and a back-scattering angle of 173° was used to perform DLS experiments. LPS and peptides were dissolved in 10mM phosphate buffer (pH 7.4), filtered and degassed. LPS was titrated with increasing concentrations of the peptide solution, in a low-volume disposable cuvette. The viscosity and refractive index of 10 mM phosphate buffer (pH 7.4) was set at 0.8924 and 1.330, respectively for data analysis. Autocorrelation data was used to generate the particle size distributions from the non-negative least squares fit algorithm provided in the instrument. Similar experiments were performed using LPS bicelles. DLS was performed in triplicates and three independent experimental sets were recorded.

### *1.10. $^{31}P$ solution state NMR of LPS*

NMR experiments were performed at 25 °C on a Bruker Avance III 500 MHz NMR spectrometer, equipped with a 5 mm SMART probe. Both LPS and peptides were dissolved in HEPES buffer supplemented with 10 % $D_2O$ at pH 4.5. The interaction of LPS with the peptides was monitored by 1D proton NMR spectra monitoring the line broadening effect LPS in the presence of increasing concentrations of peptides. A series of 1D proton decoupled $^{31}P$ NMR spectra of 0.5 mM LPS alone and with increasing concentrations of peptides, with and without 0.1 mM $MnCl_2$ as a paramagnetic quencher, were recorded with 1024 scans.

### *1.11. Calcein leakage assay*

Dye leakage assay was performed using a protocol published elsewhere (Domadia et al., 2010). Briefly, 1-palmitoyl, 2-oleoyl-phosphatidylethanolamine (POPE) and 1-palmitoyl,2-oleoyl-phosphatidylglycerol (POPG) (Avanti Polar Lipids, Alabaster, AL) were mixed to a final ratio of 7:3 (bacterial outer membrane mimic) and 3:1 (bacterial inner membrane mimic) in chloroform to obtain a stock solution of 25mg/ml each. Similarly, 1-palmitoyl-2-oleoyl-sn- glycero -3-phosphocholine (POPC), POPE and Stigmasterol were mixed in the ratio 5:4:3 (plant membrane mimic) in chloroform. All the mixtures were dried by subjecting to nitrogen gas, followed by lyophilization to generate lipid films.10 mM Tris buffer (pH 7.4), containing 70 mM calcein was added to the lipid films, followed by vigorous vortexing for 30 min. The suspension was then subjected to five freeze-thaw cycles in liquid nitrogen to obtain calcein entrapped vesicles. Passing the suspension through a mini extruder (Avanti Polar Lipids, Alabaster, AL) using stacked 100 nm pore size polycarbonate membrane filters 23 times generated large unilamellar vesicles (LUVs). Unencapsulated calcein was removed by passing the vesicles through a gel filtration based hydrated Centrisep- Spin Column. Calcein leakage was monitored using Hitachi F-7000FL spectrophotometer (slit width: 2.5 nm). The excitation and emission maxima were set at 495nm and 519nm respectively. VR18 and KG18 were titrated into a 10μM LUVs (after allowing the

stabilization of the LUVs) in 10 mM Tris containing 100 mM NaCl at pH 5.4, 6.4, 7.4, and 8.0 and enhancement in fluorescence was recorded. 0.1% Triton X-100 served as positive control.

Percentage leakage was calculated using the equation:

$$\% \text{ dye leakage} = [(F-F_0)/(F_T-F_0)] \times 100\% \quad (2)$$

where, $F_o$, F, $F_T$ denotes the basal fluorescence intensity, florescence intensity after peptide addition and maximum florescence intensity obtained after addition of 0.1% Triton X-100, respectively.

### *1.12. Liposome and LPS bicelle pull down assay*

Liposome and LPS bicelles pull down assay was performed as mentioned previously (Phan et al., 2015). Briefly, liposomes and LPS bicelles were generated as mentioned above. After washing with 10mM phosphate buffer (pH 7.4), the liposomes and LPS bicelles were incubated with 5 µg of the peptides for 30 mins. After centrifugation at 13000 rpm for 10 min, the pellet (bound fraction) and supernatant (unbound fraction) were separated and analyzed by Tricine-SDS- PAGE followed by Coomassie blue staining (Schägger, 2006). The intensity of protein bands was determined by densitometric analysis using ImageJ software. Normalization was done against appropriate protein loading control.

### *1.13. $^{31}$P solid state NMR*

Agilent NMR spectrometer (DD2) functioning at the resonance frequency of 283.31MHz for $^{31}$P and 699.88MHz for $^{1}$H, equipped with a 4mm MAS HXY Solid Probe was used to record the $^{31}$P solid state NMR experiments. Multilamellar vesicles (MLVs) were prepared by dissolving POPE and POPG in chloroform to prepare a 5 mg/ml stock to obtain a final ratio of 7:3 (mimicking bacterial outer membrane) and 3:1 (mimicking bacterial inner membrane) (Szoka et al., 1978). Similarly, POPC, POPE and Stigmasterol were mixed in the ratio 5:4:3 (to generate plant membrane mimic) in chloroform. The lipid mixtures were dried under a stream of nitrogen gas and lyophilized overnight. The lipid film was dissolved in Tris buffer (pH 7.4), allowed to hydrate for 1h by vortexing intermittently and freeze- thawed five times.

NMR experiment was set using single 90º pulse and 24 kHz TPPM proton decoupling. The π/2 pulse length was set at 6.8µs for the $^{31}$P nucleus. MLVs were kept in a 4 mm Pyrex glass tube, which was fit into the MAS probe, and sealed with parafilm. The sample temperature was maintained at 25 ºC using an Agilent temperature control unit. $^{31}$P spectra were collected with 256 scans with a cycle delay of 2 s and referenced externally to 85% phosphoric acid (0 ppm). Spectra processing was done with MestReNova software (Ver8.1) with 250 Hz line broadening.

### *1.14. Solution- state NMR*

700 MHz NMR spectrometer equipped with a 5 mm RT probe was used for NMR experiments. Briefly, NMR samples were prepared in 10% deuterated buffer (pH 4.5) and 3-Trimethylsilyl) propionic-2,2,3,3-$d_4$ acid (TSP) sodium salt was used as an internal standard. Two-dimensional $^1H$-$^1H$ total correlation spectroscopy (2D TOCSY) and two-dimensional $^1H$-$^1H$ Nuclear Over Hauser Spectroscopy (2D NOESY) were recorded for both VR18 and KG18 peptides with a mixing time of 80 ms and 150 ms, respectively. Spectral width of 12 ppm was set in both directions. The number of scans were fixed to 20 and 160 for TOCSY and NOESY, respectively. The recycle delay (D1) for both the experiments was set to 1.5 s with 456 increments in the $t_1$, and 2048 data points in the $t_2$ dimensions along with states time proportional phase incrementation (TPPI) for quadrature detection in $t_1$ dimension and excitation-sculpting scheme for water suppression for both the peptides. The peptides were subjected to successive titration with *P. aeruginosa* LPS bicelles and the change in spectra were monitored by $^1H$ NMR, acquired using an excitation-sculpting scheme for water suppression and the States-TPPI for quadrature detection in the $t_1$ dimension (Roumestand & Canet, 2000). 2D TOCSY and transferred NOESY (trNOESY) spectra of the peptide in the presence of LPS bicelles were acquired with 80 ms and 150 ms mixing time, respectively. Topspin™ v3.1 software (Bruker Biospin, Switzerland) and Sparky software was used for data acquisition and analysis.

### *1.15. NMR- derived structure calculation*

The three-dimensional structure LPS bicelle- bound peptide was calculated. Depending on the intensities in the trNOESY spectra, the volume integrals of the respective NOE cross-peaks were qualitatively differentiated into strong, medium and weak with inter-proton upper bound distances of ≤ 3.0, 3.5- 4.0 and 4.0-5.0Å, respectively, while the lower bound distance was fixed to 2.0 Å. For all non- glycine residues, the backbone dihedral angles of the peptides, phi (φ) and psi (ψ) were kept flexible (- 30° to 120° and 120° to -120°, respectively) to limit the conformational space. All structure calculations were done using CYANA program v2.1 and iterative refinement of the structure based on distance violation was performed. All hydrogen bonding constraints were eliminated from structure calculation. The NMR-derived ensemble structures were analyzed using PyMOL and MOLMOL software and their stereochemistry was checked using Procheck.

### *1.16. Molecular Dynamics Simulation*

All atom classical molecular dynamics simulations were performed in two-step to develop a molecular level understanding of the interactions of VR18 and KG18 peptides with the LPS containing bacterial membrane. In the first step, we constructed a symmetric E.coli ReLPS (Brandner et al., 2024, Berglund et al., 2015) lipid (*E.coli* lipid A + 2 Kdo sugars) bilayer consisting a total of 48 ReLPS lipids (24 lipids in each inner and outer leaflets) with initial box size of

$67.5 \times 67.5 \times 101.23\,\text{Å}^3$ using the *Bilayer Builder* in CHARMM-GUI (Jo et al., 2008, Lee et al., 2016, Lee et al., 2019, Wu et al., 2014). The bilayer was parameterized using CHARMM36m(Huang et al., 2017) force filed. Charges of the phosphate groups in the glucosamine dimer of lipid A were taken as -2e. 144 $Ca^{2+}$ ions were added in the ReLPS headgroup and core regions to neutralize the charges. The system was solvated with 7333 TIP3P(Mark & Nilsson, 2001) waters, 19 $K^+$ and 19 $Cl^-$ ions to maintain 0.15 M KCl concentration. The system was energy minimized and equilibrated in NPT ensemble at 318K temperature and 1 atm pressure. Production simulation of 1000 ns was performed in NPT ensemble and the corresponding area per lipid (Smith et al., 2019) was monitored for convergence check. Area per lipid was converged to the previously reported value (Gao et al., 2021) for this symmetric ReLPS bilayer (see Figure S1). In the next step, VR18 and KG18 peptides were modelled using CHARMM36m force filed, and one peptide of each type was placed around 1 nm distance on the top of the converged and equilibrated ReLPS bilayer structure. Initial box sizes were $63 \times 63 \times 118\,\text{Å}^3$. 144 $Ca^{2+}$ ions were still present in the ReLPS headgroup and core regions to neutralize the charges. Besides, both the systems were solvated using ~ 9000 TIP3P waters, 42 $K^+$ and 48 $Cl^-$ ions to maintain 0.15 M KCl concentration. These two systems were taken as the starting configurations for the simulations of the bilayer-peptide systems. The two systems were energy minimized and equilibrated in NPT ensemble at 318 K temperature and 1 atm pressure. Production simulations of 800 ns were performed in NPT ensemble.

All the above-mentioned simulations were performed using GROMACS (Abraham et al., 2015) v2021.5 and v2024.3 under periodic boundary conditions. Energy minimizations were done using the steepest descent method. Temperature and pressure were controlled using velocity rescale thermostat(Bussi et al., 2007) and Parrinello-Rahman barostat (Anonymous, 1981) with time constants of 1 ps and 5 ps respectively. Long range electrostatic interactions were handled using particle mesh Ewald (PME) (Essmann et al., 1995) summation method. Cut-off distances for electrostatic and van der Waals interactions were set to $12\,\text{Å}$. Bonds containing hydrogen atoms were constrained using LINCS (Hess et al., 1997) algorithm.

### *1.17. PCR to confirm integration of empty pCAMBIA 1304 in vector control plants*

Total DNA was isolated from vector control leaves using Plant DNAzol reagent (Invitrogen) following the manufacturer's protocol. Briefly, leaf tissues were crushed in liquid nitrogen, and the powdered tissues were suspended in Plant DNAzol reagent (0.3 ml/0.1 g plant tissue) supplemented with RNAse A (100 µg RNAse A/ml Plant DNAzol), mixed by gentle inversion, and incubated at 25 °C under shaking conditions for 5 min. 0.3 ml chloroform was added to the mixture, and centrifuged at 12,000 g for 10 min. DNA was precipitated with 100 % cold ethanol from the aqueous phase and finally resuspended in TE buffer (10 mM Tris–HCl, pH 8.0, and 1

mM EDTA). PCR was carried out for *hptII* specific gene. Amplification of specific product was confirmed by 2% agarose gel electrophoresis of the PCR end- products.

### *1.18. Identification of stress genes in tobacco and primer designing*

The SOL Genomics Network (SGN, https://solgenomics.net/) database (ITAG release 2.0), Ensembl Plants (https://plants.ensembl.org/) (Genome assembly: SL3.0), and NCBI nucleotide database were searched for *N. tabacum* stress genes. Primers were designed manually and analysed using the OligoAnalyzer™ software (https://www.idtdna.com/pages/tools/oligoanalyzer) to determine their specificity, G-C content, melting temperature, and dimerization properties (Table S2).

**Table S1**. Prediction of allergenicity of VR18 and KG18 based on SVM method using amino acid composition of the peptides, to obtain comprehensive information like score, threshold, positive predictive value (PPV), negative predictive value (NPV) and distance from threshold of the prediction. Any value of PPV >80% is indicative of the protein being an allergen.

| Peptide | Sequence | Allergenicity | Score | Default Threshold | Positive predictive value (%) | Negative Predictive value (%) |
|---|---|---|---|---|---|---|
| VR18 | VARGWGRKFPLFGKNKSR | Non- allergen | -0.58 | -0.4 | 18.21 | 71.24 |
| KG18 | KNKSRVARGWCRKCPLFG | Non- allergen | -0.63 | -0.4 | 22.82 | 92.94 |

**Table S2**. List of primers used in this study.

| Name | Primer Sequence (5' to 3') | Orientation | Detection/Usage |
|---|---|---|---|
| *VR18*- F | CAGCTTTCGTTCTTATCTTGTTCG | Forward | RT-PCR Amplification of *VR18* gene to check its expression from cDNA |
| *VR18*- R | GGACACTTCCTACCCCATC | Reverse | RT-PCR Amplification of *VR18* gene to check its expression from cDNA |
| *KG18*- F | ATGGTGAACAGATCAGTGGC | Forward | RT-PCR Amplification of *KG18* gene to check its expression from cDNA |
| *KG18*- R | CATCCCCTAGCAACTCTTGAC | Reverse | RT-PCR Amplification of *KG18* gene to check its expression from cDNA |
| *PR1a*- F | GGACGACCAGGTAGCAGC | Forward | RT-PCR Amplification of *PR1a* gene to check its expression from cDNA |
| *PR1a*- R | AATCGCCACTTCCCTCAGC | Reverse | RT-PCR Amplification of *PR1a* gene to check its expression from cDNA |
| *ABA2*- F | GAGGATGGACAGCAGTATTCAGG | Forward | RT-PCR Amplification of *ABA2* gene to check its expression from cDNA |
| *ABA2*- R | CCAGTGTAACAAGTGTAGCCAGAG | Reverse | RT-PCR Amplification of *ABA2* gene to check its expression from cDNA |
| *HptII*- F | GATGTTGGCGACCTCGTATT | Forward | Transgenic confirmation |
| *HptII*- R | GAGTTTAGCGAGAGCCTGACCTAT | Reverse | Transgenic confirmation |
| *EF1*α F | TGATCTGCTGCTGTAACAAGATGG | Forward | RT-PCR amplification of *EF1*α gene from cDNA |
| *EF1*α R | GTCAAGAGCCTCAAGGAGGGTTG | Reverse | RT-PCR amplification of *EF1*α gene from cDNA |

**Table S3**. Summary of the structural statistics for the 20 lowest energy ensemble structures of VR18 and KG18 in presence of *P.aeruginosa* LPS bicelle.

| | *Pseudomonas aeruginosa* Lipopolysaccharide Bicelle | |
|---|---|---|
| | **VR18** | **KG18** |
| **Distance restraints** | | |
| Intra residue (i - j = 0) | 29 | 26 |
| Sequential (\|i - j\| = 1) | 51 | 58 |
| Medium range (2 ≤ \|i - j\| ≤ 4) | 18 | 10 |
| Long range (\|i - j\| ≥ 5) | 8 | 13 |
| Total | **114** | **107** |
| **Angular restraints** | | |
| Φ | 16 | 16 |
| Ψ | 15 | 15 |
| **Distance restraints from violation** (≥ 0.4 Å) | 1 | 1 |
| **Deviation from mean structure (Å)** | | |
| Average backbone to mean structure | 1.06 ± 0.38 Å | 1.31 ± 0.50 Å |
| Average heavy atom to mean structure | 1.95 ± 0.56 Å | 2.21 ± 0.75 Å |
| **% Residues in Ramachandran plot*** | | |
| most favoured region | 58.3 | 61.5 |
| additionally allowed region | 41.7 | 38.5 |
| generously allowed region | 0.0 | 0.0 |
| disallowed region | 0.0 | 0.0 |

* Procheck NMR based analysis (https://services.mbi.ucla.edu/PROCHECK/)

**Table S4**. List of metabolites identified in control and transgenic plants using LC-ESI-QTOF MS/MS given in a separate excel sheet.

**Table S5**. Raw peak intensity file for Control and VR18 groups given in a separate excel sheet.

**Table S6**. Raw peak intensity file for Control and KG18 groups given in a separate excel sheet.

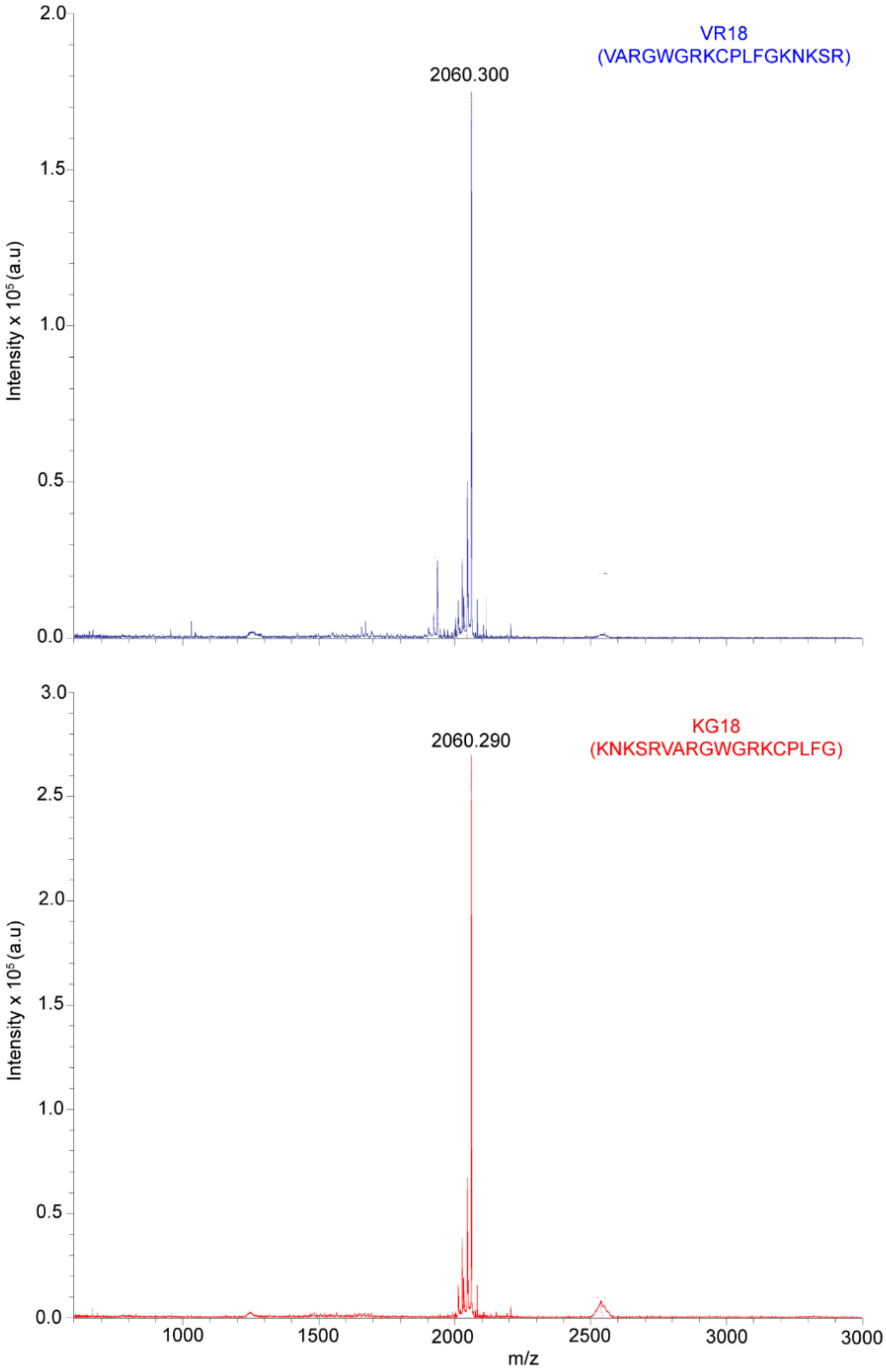


**Fig. S1**. MALDI analysis of VR18 and KG18 peptide (Bruker ATS-00699, autoflex TOF/TOF).

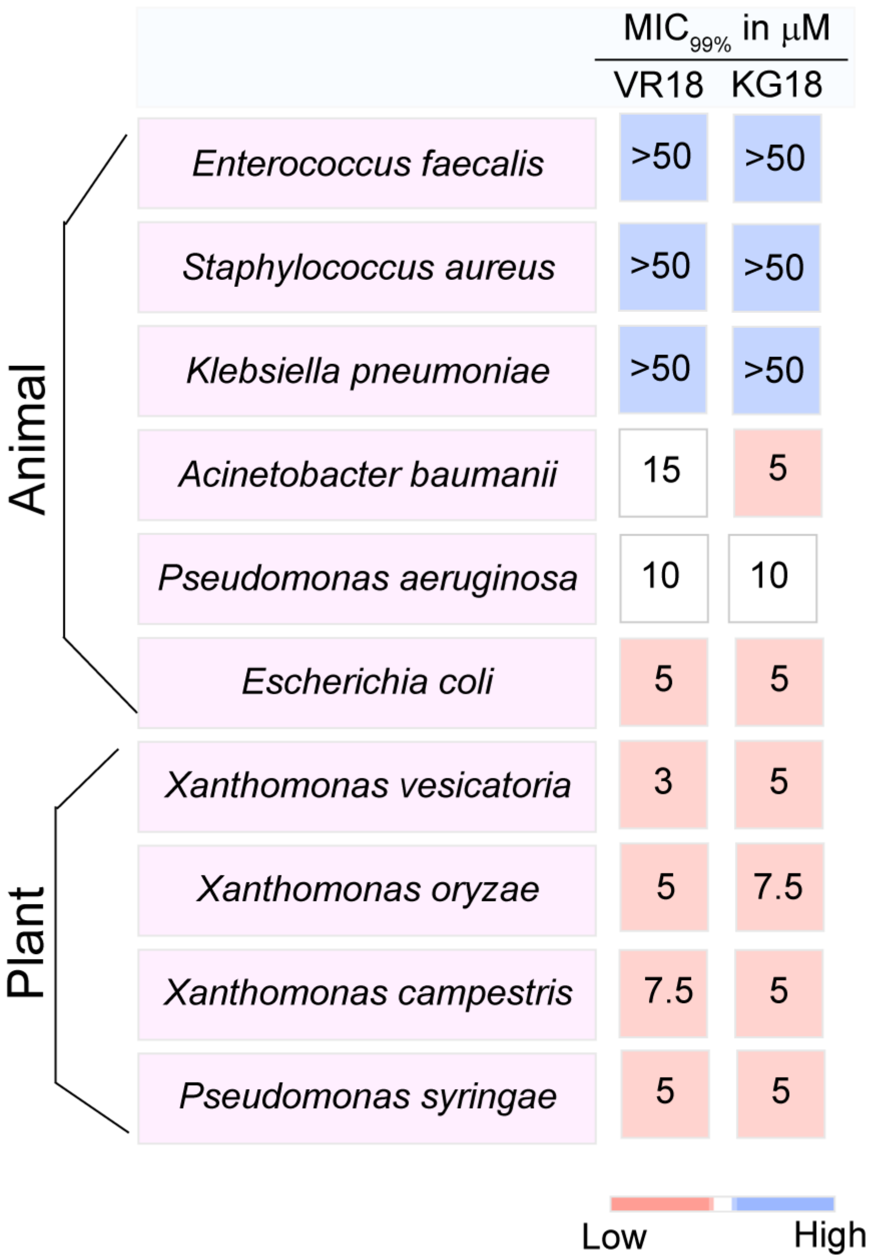


**Fig. S2**. $MIC_{99\%}$ values of VR18 and KG18 against animal (ESKAPE) as well as plant pathogens.

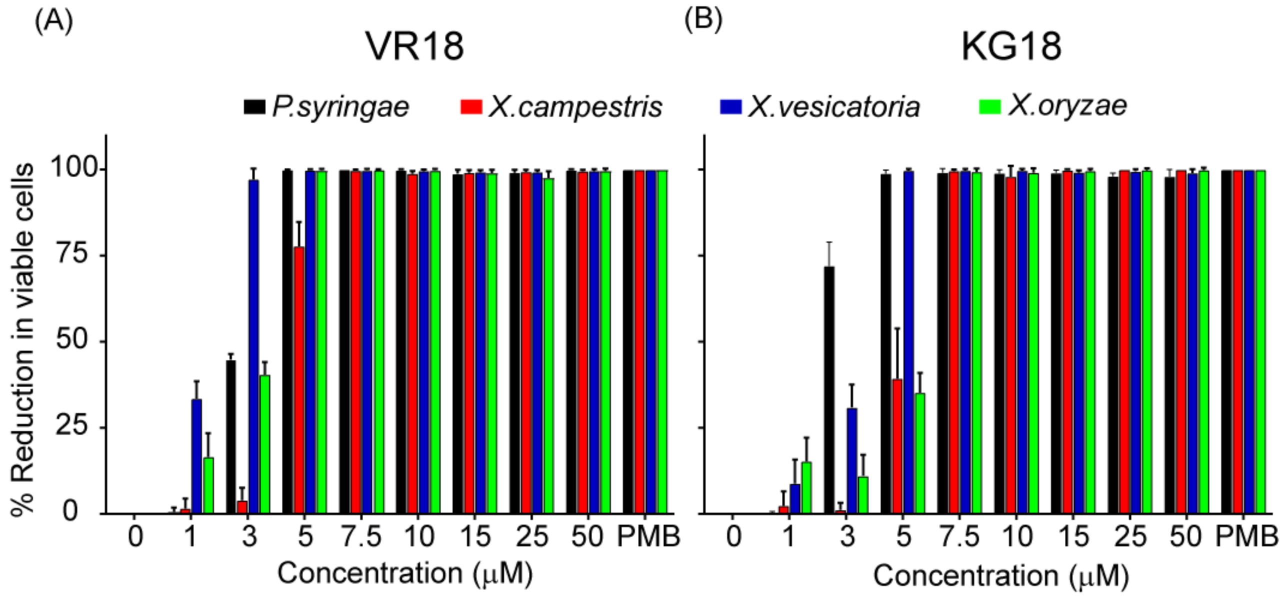


**Fig. S3**. $MIC_{99\%}$ of VR18 and KG18 against plant pathogens. Microbroth dilution assay was performed in the presence of increasing concentrations of (A) VR18 and (B) KG18. Treatment with both the peptides showed growth inhibition of the plant pathogens at low concentrations. Peptide untreated cells served as negative control and cells treated with polymyxin B (PMB) served as positive control. After normalization, the percentage reduction in the viable cells was calculated and the concentration depicting 99% reduction in the viable cell count was represented in the form of $MIC_{99\%}$.

(A) (B)

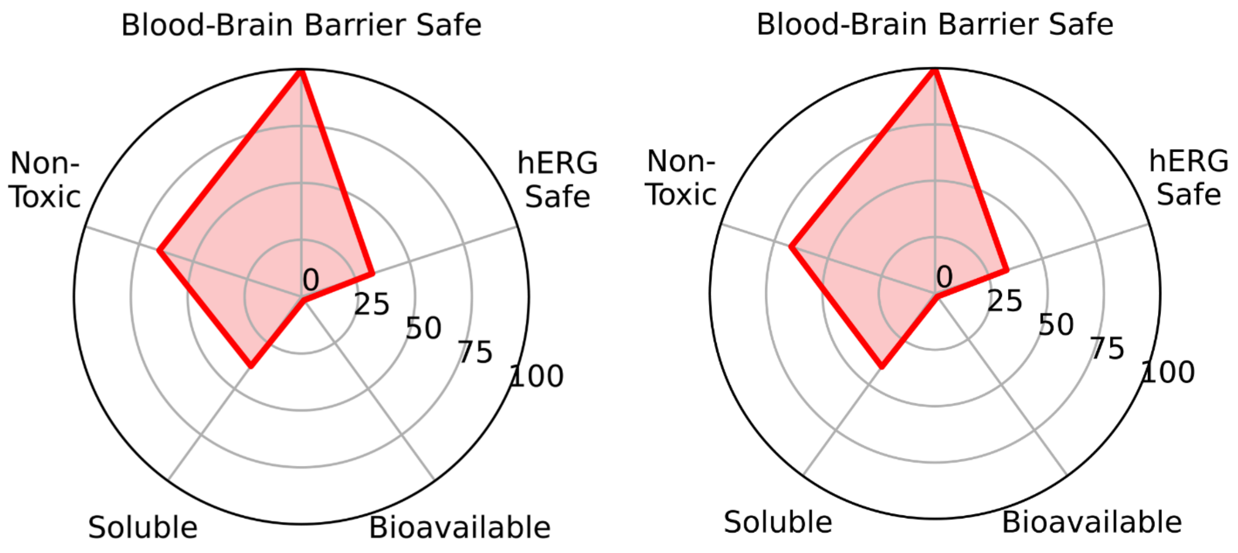


**Fig. S4**. ADMET property prediction profiles of VR18 (A) and KG18 (B) suggesting the low blood brain barrier penetration / hERG toxicity indicating their lack of toxicity in human. This analysis was carried out using ADMET-AI machine learning server (https://admet.ai.greenstonebio.com).

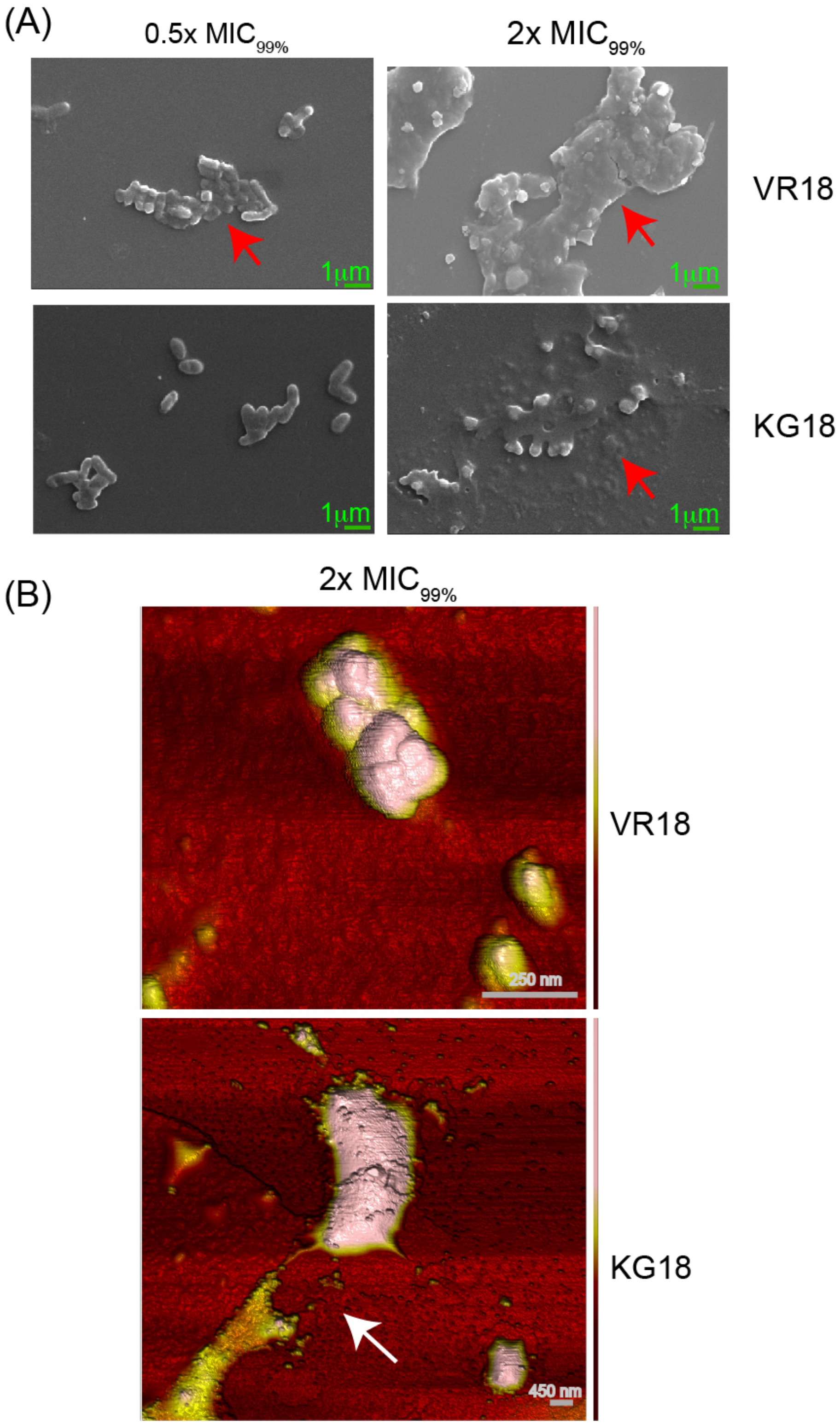


**Fig. S5**. Microscopic analysis of the effect of VR18 and KG18 on *P. syringae* cells. (A) SEM images of *P. syringae* treated with VR18 and KG18. At 0.5x $MIC_{99\%}$ concentration, cell clumping and initiation of membrane disruption was observed, whereas at 2x $MIC_{99\%}$ concentration, there was complete loss of membrane integrity. (B) AFM images of *P. syringae* treated with VR18 and KG18 at 2x $MIC_{99\%}$ concentration showing membrane deformations.

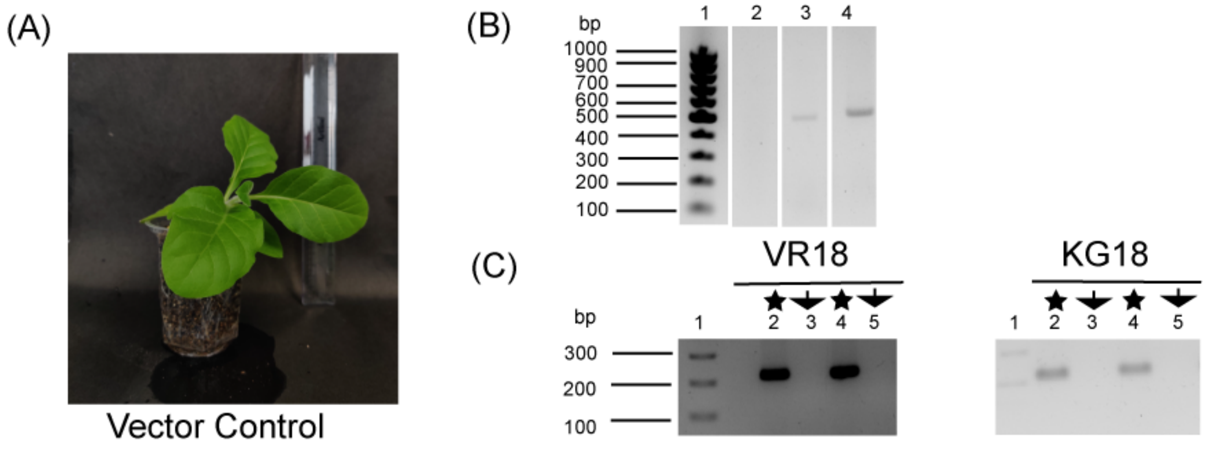


**Fig. S6**. Expression of pCAMBIA 1304 vector on *Nicotiana tabacum* to generate vector control plants. (A) Photographic image of 1 month old vector control plant. (B) Agarose gel photograph showing amplification of *hpt*II gene (440 bp) from cDNA of two vector control lines (lane 3 and 4). In wild-type plant there was no amplification of *hpt*II gene (lane 2). (C) Reverse Transcription PCR of VR18 and KG18 gene in vector control plants. Agarose gel image depicts that there was no amplification of VR18 or KG18 gene in the two lines of vector control plants. EF1α gene was used as loading control. EF1α expression is denoted by stars and expression of VR18 or KG18 is expressed by downward arrow.

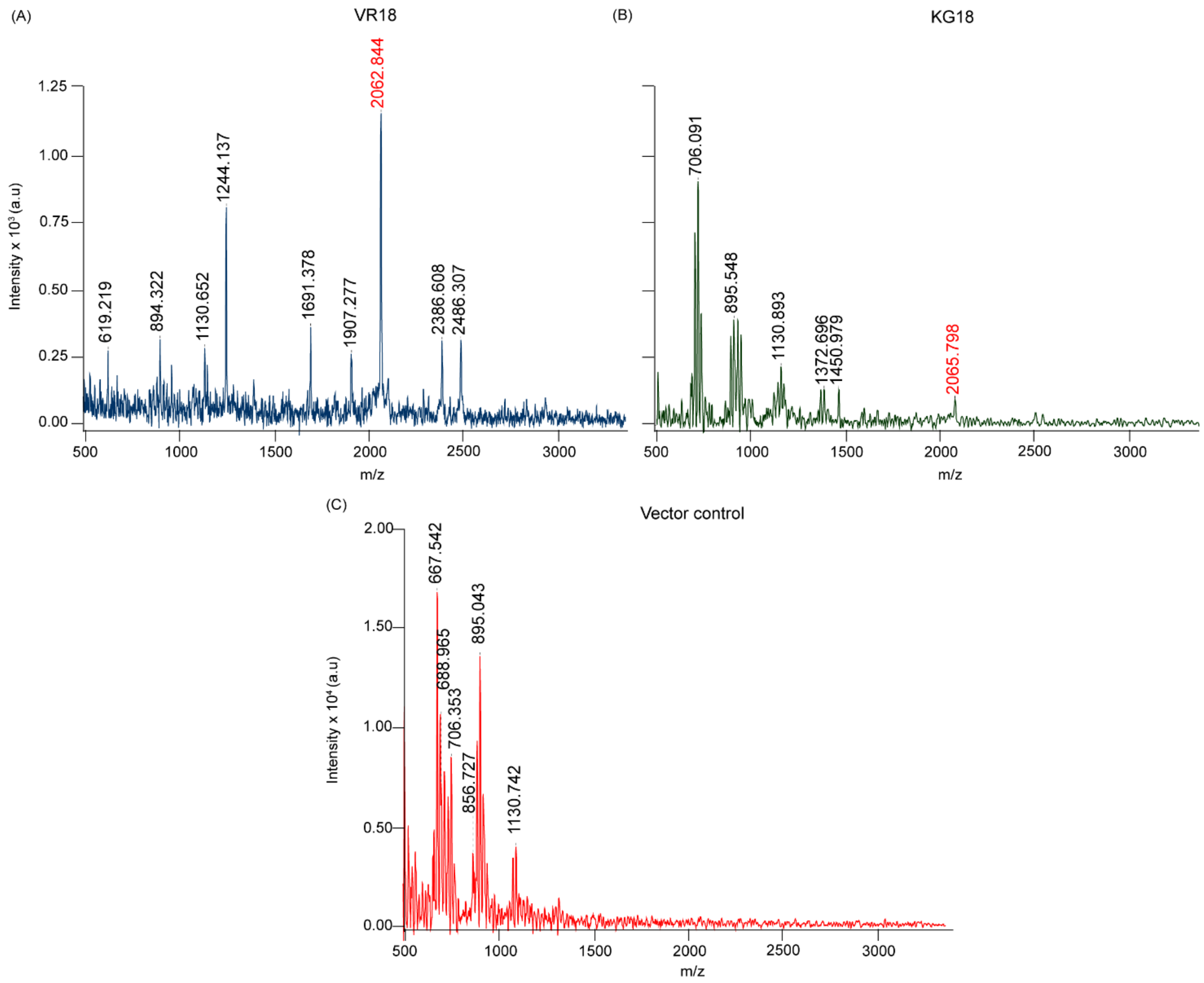


**Fig. S7**. MALDI analysis of the plant extracts from transgenic as well as vector control plants. (A) Spectra of the extracts from VR18 expressing plants shows a peak in the m/z of 2063. (B) Spectra of the extracts from KG18 expressing plants shows a peak in the m/z of 2066. (C) Spectra of the extracts from vector control plants did not show any peak in the m/z range corresponding to the molecular weight of the peptides.

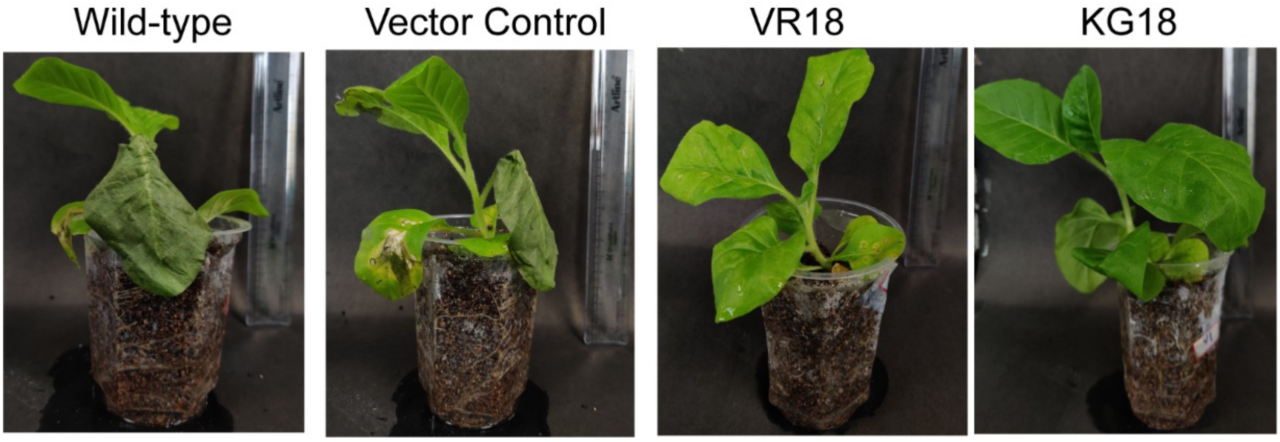


**Fig. S8.** *In vivo* analysis of the antibacterial activity of VR18 and KG18 against *Pseudomonas syringae* pv. tabaci. Disease symptoms recorded after 96 h. Photograph of leaves from infected wild type and vector control show extensive chlorosis along with stunted growth. While comparatively mild symptoms were observed in case of VR18 and KG18 expressing plants post 96 h of infection.

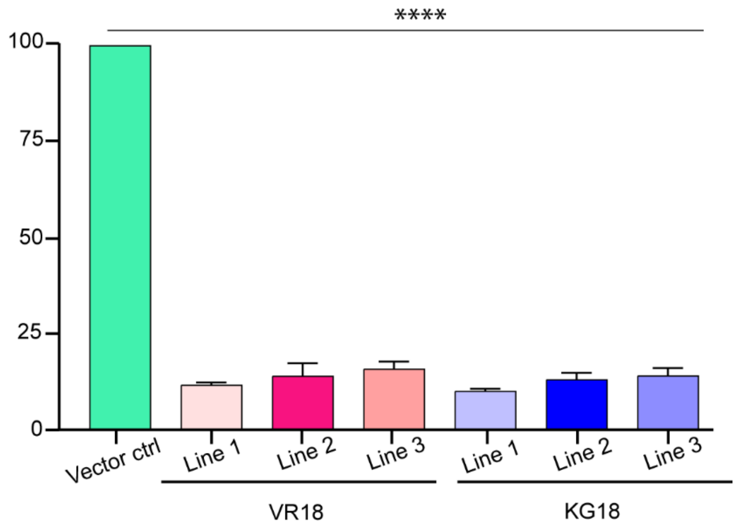


**Fig.S9.** Leaf iinfiltration assay to evaluate the anti-bacterial activity of three different transgenic lines of VR18 and KG18 expressing plants against *Pseudomonas syringae* pv. *tabaci*. After 96 h of infiltration, the CFU count was performed on King's media containing 50 μg/ml Rifampicin.

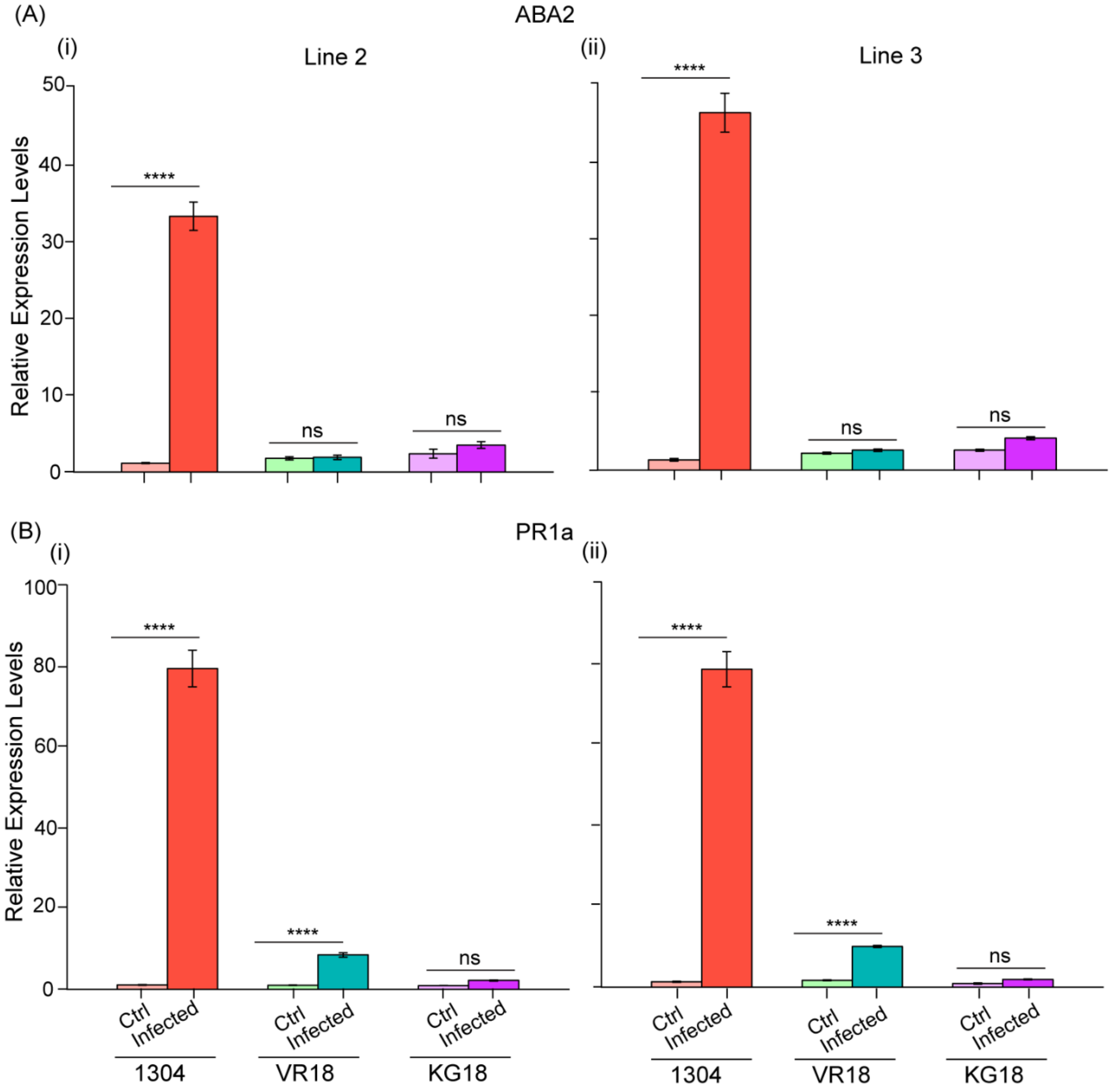


**Fig. S10.** Assessment of AMP transgenics at the molecular level in different lines of *Nicotiana tabacum* expressing VR18 and KG18 peptides. (A) Transcript levels of the abiotic stress response gene, *ABA2*, after infection with *P. syringae* were measured in (i) Line 2 and (ii) Line 3. RT-PCR analysis of RNA from 1-month-old vector control and transgenic plants showed significant upregulation of *ABA2* in control plants. (B) Transcript levels of the biotic stress response gene, *PR1a*, post-infection was also analyzed (i) Line 2 and (ii) Line 3. The RT-PCR results indicated notable upregulation of *PR1a* in control plants, while plants expressing VR18 showed only a slight increase in expression compared to the vector control. Each column represents the mean of three biological replicates, with error bars indicating SEM. Significant changes are marked with * ($p \leq 0.05$) according to the two-tailed Student's t-test, while ns indicates non-significance.

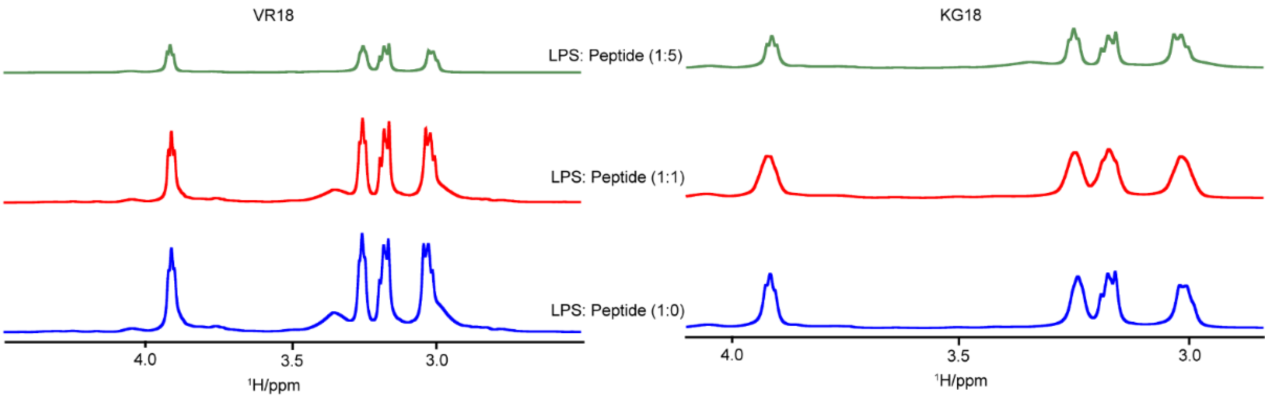


**Fig. S11.** One dimensional proton NMR spectra of acyl chains of LPS in absence and presence of VR18 and KG18. Upon peptide addition, concentration dependent line broadening and chemical shift perturbation was observed.

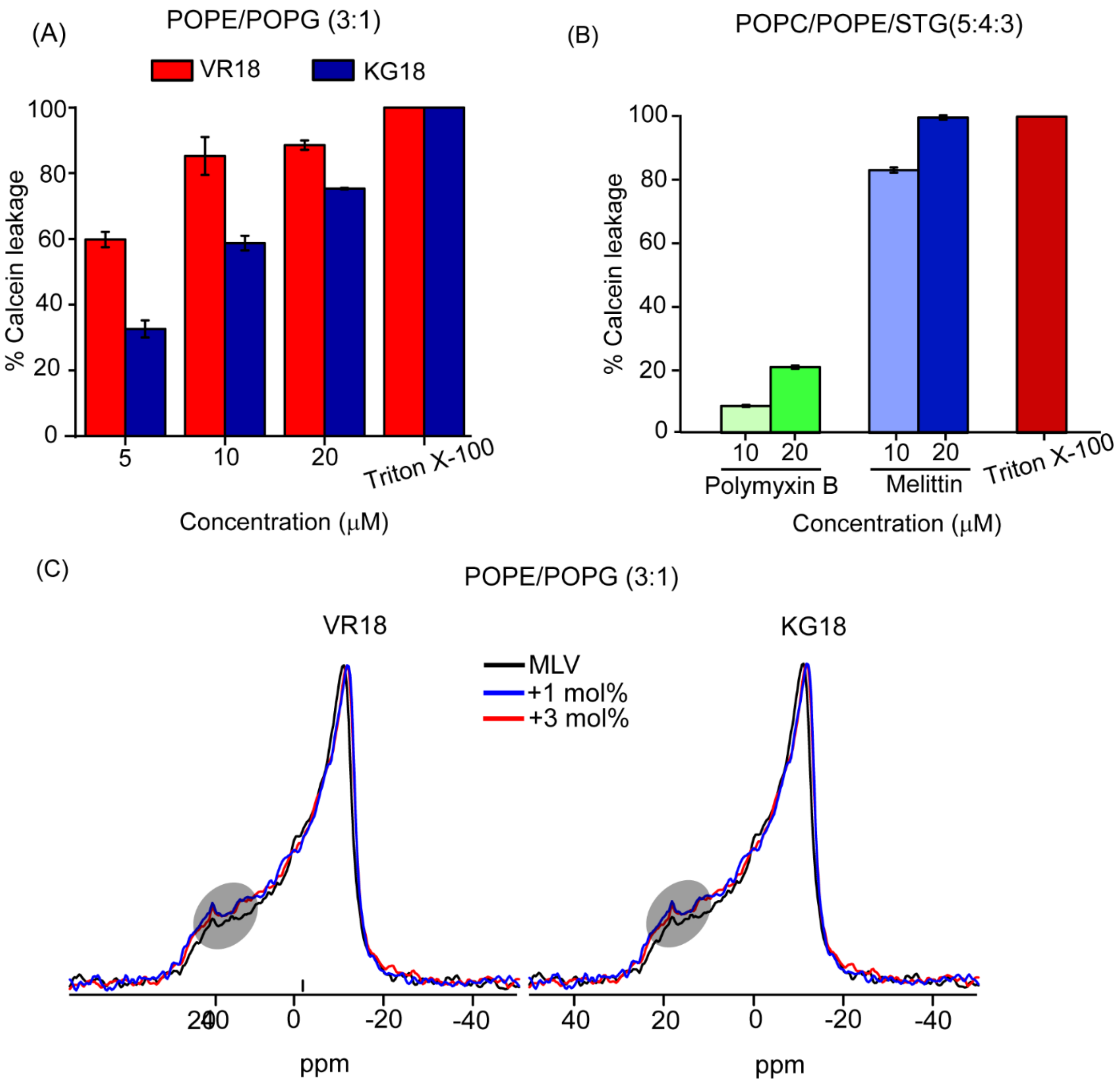


**Fig. S12**. Interaction of different peptides with membrane model mimics. (A) Calcein leakage of 3:1 POPE/POPG vesicles upon increasing concentration of VR18 and KG18. Both VR18 and KG18 caused instantaneous release of calcein at physiological conditions of pH 7.4. Triton X-100 served as positive control. (B) Calcein leakage of 5:4:3 POPC/POPE/STG vesicles upon increasing concentration of Polymyxin B and Melittin. Both Polymyxin B and Melittin caused instantaneous release of calcein upto 22 % and 99 %, respectively, at physiological conditions (pH 7.4). Triton X-100 served as positive control. (C) $^{31}$P NMR of 3:1 POPE/POPG and multi lamellar vesicles (MLVs) in absence and presence of VR18 and KG18. Increasing concentration of both peptides showed spectral thickening (highlighted) as well as increased spectral span in 3:1 POPE/POPG vesicles.

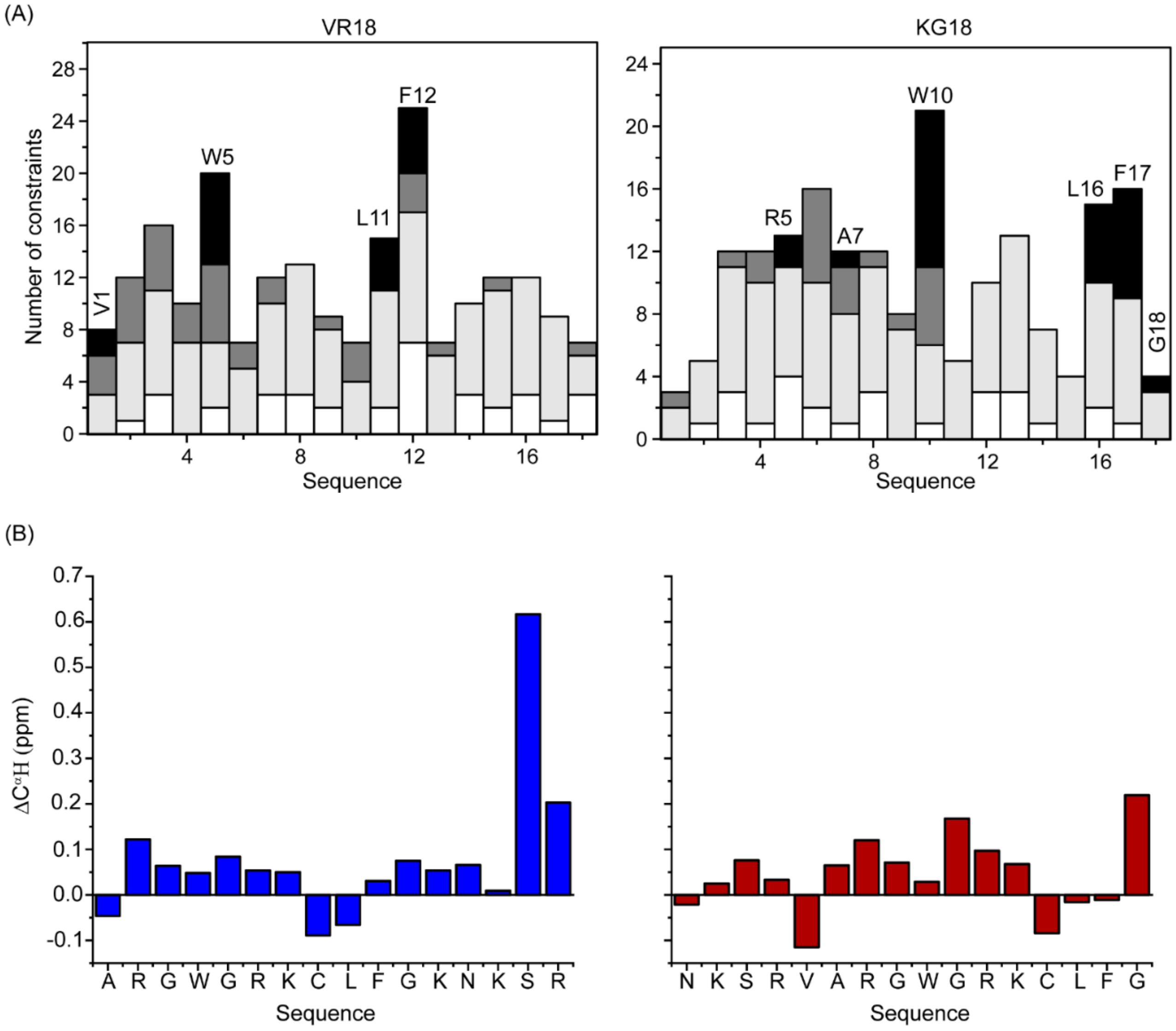


**Fig. S13**. (A) Histogram plot showing sequence- wise NOE connectivity in the LPS bicelles bound conformation of VR18 and KG18. (B) Chemical shift plot of bound VR18 and KG18. There was no significant signature for α-helical or β-sheet structure, implying that the peptide preferably assumes a loop structure in the bound conformation.

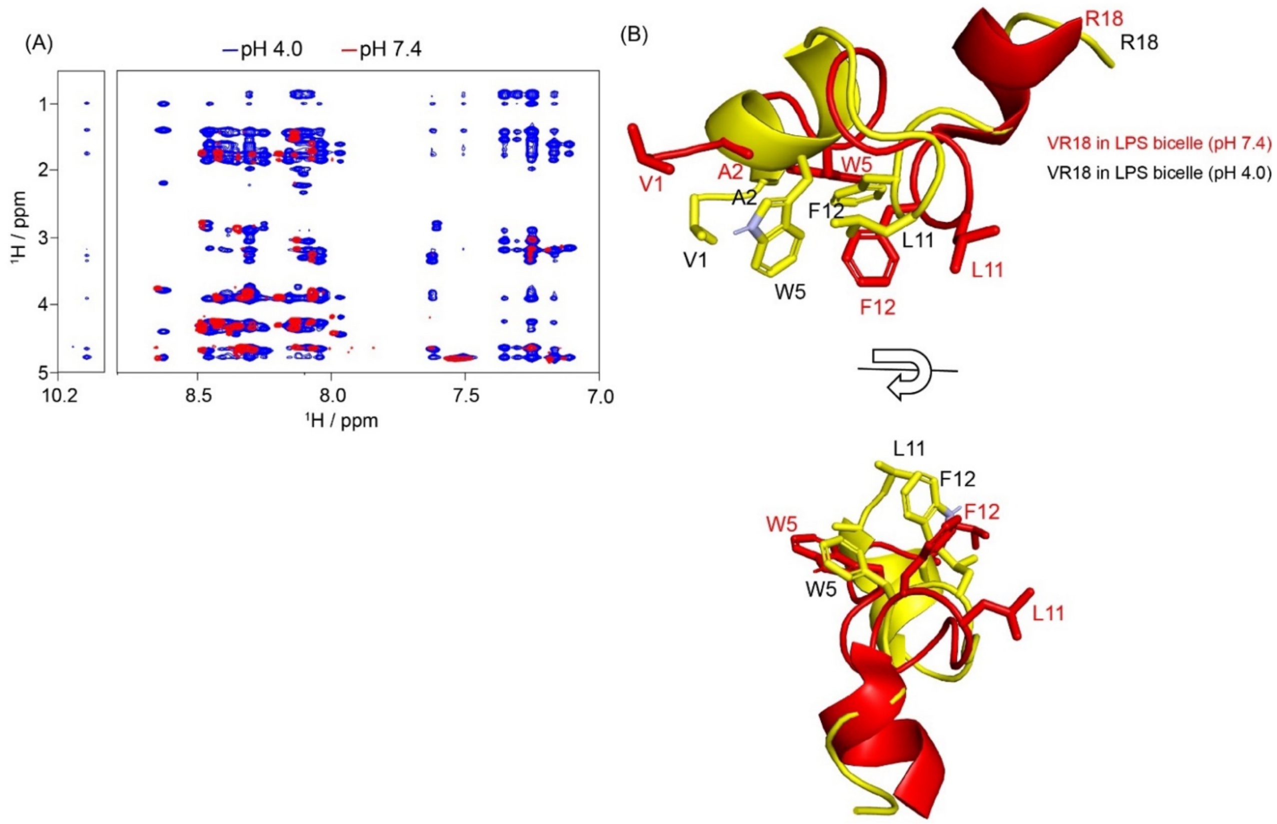


**Fig. S14**. (A) Overlay of 2D *tr*NOESY spectra of VR18 in presence of LPS bicelles at pH 4.0 and 7.4. Greater number of NOE cross-peaks were observed at pH 4.0 compared to pH 7.4. (B) Structural comparison of LPS bicelle bound VR18 at pH 4.0 (yellow colour) and 7.4 (red colour), respectively.

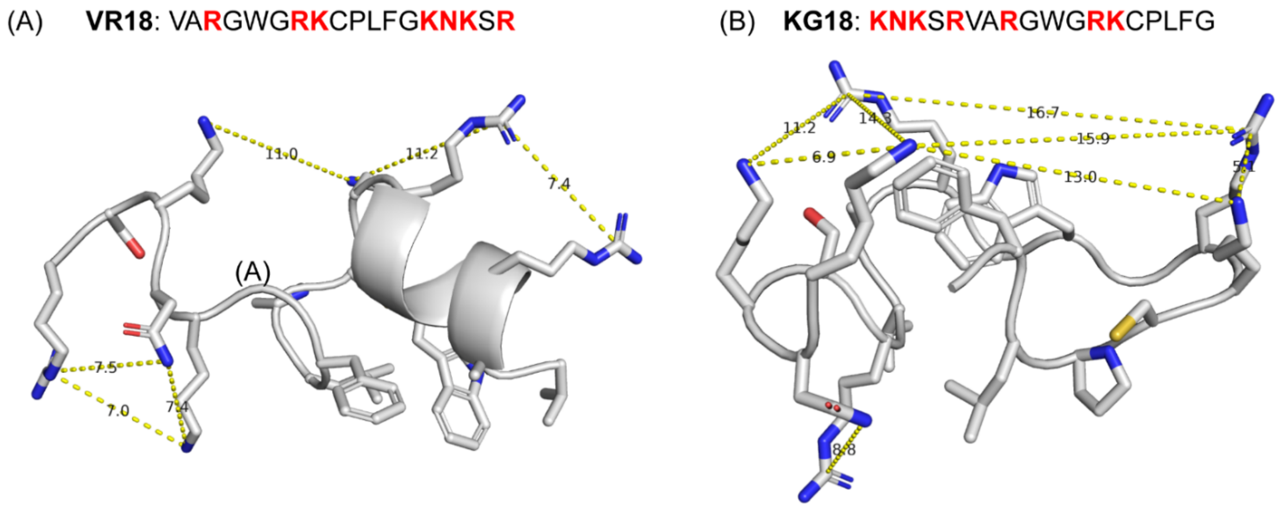


**Fig. S15**. Cartoon representation of a single molecule of VR18 (A) and KG18 (B) bound to LPS bicelles, highlighting the distance between the head group of positively charged residues, Arg and Lys. These hydrophilic head groups maintained a clear separation of 11-17 Å or < 10 Å, comparable to the LPS phosphate head groups. The distance between the head groups was calculated using PyMol.

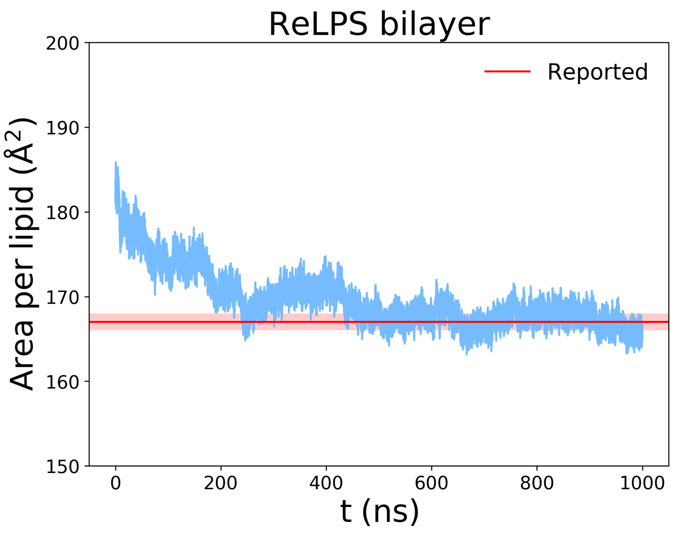


**Fig. S16.** Area per lipid for the symmetric ReLPS bilayer is shown as a function of simulation time. Red shaded region represents the error bar of the reported value.

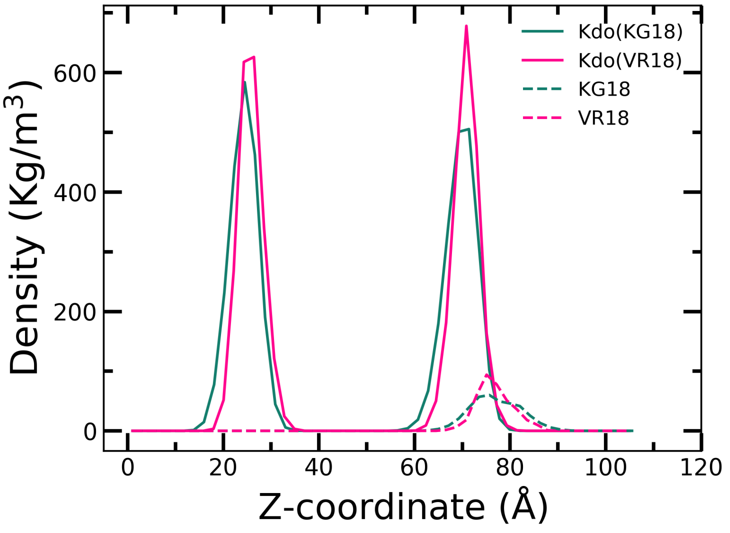


**Fig. S17.** Density profiles of the Kdo sugar head groups on the peptide bound bilayer leaflet and the peptides along the Z-axis.

***References***

Abraham MJ, Murtola T, Schulz R, Pall S, Smith JC, Hess B, Lindahl E, 2015. GROMACS: High performance molecular simulations through multi-level parallelism from laptops to supercomputers. *SoftwareX* **1-2**, 19-25.

Berglund NA, Piggot TJ, Jefferies D, Sessions RB, Bond PJ, Khalid S, 2015. Interaction of the antimicrobial peptide polymyxin B1 with both membranes of E. coli: a molecular dynamics study. *PLoS Comput Biol.* **11**, e1004180.

Brandner AF, Prakaash D, Blanco González A, Waterhouse F, Khalid S, 2024. Faster but Not Sweeter: A Model of Escherichia coli Re-level Lipopolysaccharide for Martini 3 and a Martini 2 Version with Accelerated Kinetics. *J Chem Theory Comput* **20**, 6890-903.

Bussi G, Donadio D, Parrinello M, 2007. Canonical sampling through velocity rescaling. *J Chem Phys.* **126**.

Domadia PN, Bhunia A, Ramamoorthy A,Bhattacharjya S, Bhattacharjya S, 2010. Structure, interactions, and antibacterial activities of MSI-594 derived mutant peptide MSI-594F5A in lipopolysaccharide micelles: role of the helical hairpin conformation in outer-membrane permeabilization. *J Am Cheml Soc.* **132(51)**, 18417-28.

Essmann U, Perera L, Berkowitz ML, Darden T, Lee H, Pedersen LG, 1995. A smooth particle mesh Ewald method. *J Chem Phys.* **103**, 8577-93.

Gao Y, Lee J, Smith IPS, Lee H, Kim S, Qi Y, Klauda JB, Widmalm G, Khalid S, Im W, 2021. CHARMM-GUI Supports Hydrogen Mass Repartitioning and Different Protonation States of Phosphates in Lipopolysaccharides. *J Chem Inf Model* **61**, 831-9.

Hess B, Bekker H, Berendsen HJC, Fraaije JGEM, 1997. LINCS: A linear constraint solver for molecular simulations. *J Comput Chem.* **18**, 1463-72.

Huang J, Rauscher S, Nawrocki G, Ran T, Feig M, de Groot BL, Grubmüller H, MacKerell AD Jr., 2017. CHARMM36m: an improved force field for folded and intrinsically disordered proteins. *Nat Methods.* **14**, 71-3.

Jo S, Kim T, Iyer VG, Im W, 2008. CHARMM-GUI: a web-based graphical user interface for CHARMM. *J Comput Chem.* **29**, 1859-65.

Lee J, Cheng X, Swails JM, Yeom MS, Eastman PK, Lemkul JA, Wei S, Buckner J, Jeong JC, Qi Y, Jo S, Pande VS, Case DA, Brooks CL 3rd, MacKerell AD Jr, Klauda JB, Im W, 2016. CHARMM-GUI Input Generator for NAMD, GROMACS, AMBER, OpenMM, and CHARMM/OpenMM Simulations Using the CHARMM36 Additive Force Field. *J Chem Theory Comput.* **12**, 405-13.

Lee J, Patel DS, Ståhle J*, et al.*, 2019. CHARMM-GUI Membrane Builder for Complex Biological Membrane Simulations with Glycolipids and Lipoglycans. *J Chem Theory Comput* **15**, 775-86.

Mark P, Nilsson L, 2001. Structure and Dynamics of the TIP3P, SPC, and SPC/E Water Models at 298 K. *J Phys Chem A* **105**, 9954-60.

Phan TK, Lay FT, Poon IKH, Hinds MG, Kvansakul M, Hulett MD, 2015. Human β-defensin 3 contains an oncolytic motif that binds PI(4,5)P2 to mediate tumour cell permeabilisation. *Oncotarget* **7**, 2054 - 69.

Roumestand C, Canet D, 2000. Extending the excitation sculpting concept for selective excitation. *J Magn Reson.* **147**, 331-339.

Saha S, Raghava GPS, 2006. AlgPred: prediction of allergenic proteins and mapping of IgE epitopes. *Nucleic Acids Res.* **34**, W202-W9.

Schägger H, 2006. Tricine–SDS-PAGE. *Nat Protoc.* **1**, 16-22.

Smith DJ, Klauda JB, Sodt AJ, 2019. Simulation Best Practices for Lipid Membranes [Article v1.0]. *Living J Comput Mol Sci* **1**.

Swanson K, Walther P, Leitz J, Mukherjee S, Wu JC, Shivnaraine RV, Zou J, 2024. ADMET-AI: a machine learning ADMET platform for evaluation of large-scale chemical libraries. *Bioinformatics* **40(7)**, btae416.
Szoka F Jr, Papahadjopoulos D, 1978. Procedure for preparation of liposomes with large internal aqueous space and high capture by reverse- phase evaporation. *Proc Natl Acad Sci USA* **75(9)**, 4194-8.
Wu EL, Cheng X, Jo S*, et al.*, 2014. CHARMM-GUI Membrane Builder toward realistic biological membrane simulations. *J Comput Chem.* **35**, 1997-2004.
